# Severity of Acute Infectious Mononucleosis Correlates with Cross-Reactive Influenza CD8 T-Cell Receptor Repertoires

**DOI:** 10.1128/mBio.01841-17

**Published:** 2017-12-05

**Authors:** Nuray Aslan, Levi B. Watkin, Anna Gil, Rabinarayan Mishra, Fransenio G. Clark, Raymond M. Welsh, Dario Ghersi, Katherine Luzuriaga, Liisa K. Selin

**Affiliations:** a Department of Pathology, University of Massachusetts Medical School, Worcester, Massachusetts, USA; b School of Interdisciplinary Informatics, University of Nebraska, Omaha, Nebraska, USA; c Program in Molecular Medicine, University of Massachusetts Medical School, Worcester, Massachusetts, USA; d Immunology and Microbiology Program, University of Massachusetts Medical School, Worcester, Massachusetts, USA; National Institute of Allergy and Infectious Diseases

**Keywords:** CD8, Epstein-Barr virus, TCR repertoire, cross-reactive, heterologous immunity, immune memory, influenza

## Abstract

Fifty years after the discovery of Epstein-Barr virus (EBV), it remains unclear how primary infection with this virus leads to massive CD8 T-cell expansion and acute infectious mononucleosis (AIM) in young adults. AIM can vary greatly in severity, from a mild transient influenza-like illness to a prolonged severe syndrome. We questioned whether expansion of a unique HLA-A2.01-restricted, cross-reactive CD8 T-cell response between influenza virus A-M1_58_ (IAV-M1) and EBV BMLF1_280_ (EBV-BM) could modulate the immune response to EBV and play a role in determining the severity of AIM in 32 college students. Only *ex vivo* total IAV-M1 and IAV-M1+EBV-BM cross-reactive tetramer^+^ frequencies directly correlated with AIM severity and were predictive of severe disease. Expansion of specific cross-reactive memory IAV-M1 T-cell receptor (TCR) Vβ repertoires correlated with levels of disease severity. There were unique profiles of qualitatively different functional responses in the cross-reactive and EBV-specific CD8 T-cell responses in each of the three groups studied, severe-AIM patients, mild-AIM patients, and seropositive persistently EBV-infected healthy donors, that may result from differences in TCR repertoire use. IAV-M1 tetramer^+^ cells were functionally cross-reactive in short-term cultures, were associated with the highest disease severity in AIM, and displayed enhanced production of gamma interferon, a cytokine that greatly amplifies immune responses, thus frequently contributing to induction of immunopathology. Altogether, these data link heterologous immunity via CD8 T-cell cross-reactivity to CD8 T-cell repertoire selection, function, and resultant disease severity in a common and important human infection. In particular, it highlights for the first time a direct link between the TCR repertoire with pathogenesis and the diversity of outcomes upon pathogen encounter.

## IMPORTANCE

The pathogenic impact of immune responses that by chance cross-react to unrelated viruses has not been established in human infections. Here, we demonstrate that the severity of acute infectious mononucleosis (AIM), an Epstein-Barr virus (EBV)-induced disease prevalent in young adults but not children, is associated with increased frequencies of T cells cross-reactive to EBV and the commonly acquired influenza A virus (IAV). The T-cell receptor (TCR) repertoire and functions of these cross-reactive T cells differed between mild- and severe-AIM patients, most likely because these two groups of patients had selected different memory TCR repertoires in response to IAV infections encountered earlier. This heterologous immunity may explain variability in disease outcome and why young adults with more-developed IAV-specific memory T-cell pools have more-severe disease than children, who have less-developed memory pools. This study provides a new framework for understanding the role of heterologous immunity in human health and disease and highlights an important developing field examining the role of T-cell repertoires in the mediation of immunopathology.

## INTRODUCTION

Over 95% of the world’s population acquires a persistent infection with Epstein-Barr virus (EBV) before the age of 30. The vast majority of acute EBV infections occur in childhood and are essentially asymptomatic or unremarkable (1, 2). However, 30 to 37% of U.S. college freshmen are EBV seronegative (3). Half of these EBV-susceptible individuals acquire EBV infection in the first 2 years of college, with most (60 to 77%) displaying symptoms of acute infectious mononucleosis (AIM) (3, 4). AIM is associated with a massive CD8 T-cell expansion; symptoms can vary greatly in severity from a mild short influenza-like illness to a more severe syndrome with sore throat, lymphadenopathy, splenomegaly, hepatomegaly, and debilitating fatigue lasting months (1, 2). A rare 5% of the population appears never to acquire the infection and remains EBV seronegative (5). Severe illness requiring hospitalization has been reported in individuals who acquire primary EBV infection late in life (6). Persistent EBV infection is also associated with the development of certain malignancies, including nasopharyngeal carcinoma and Burkitt’s lymphoma (2) or autoimmune disorders such as multiple sclerosis (MS) (7).

The exact mechanisms that lead to induction of AIM are still very poorly understood. Most prior research suggests that EBV-specific CD8 T-cell responses are robustly expanded and may contribute to immunopathology in AIM; during the persistent phase of infection, EBV-specific CD8 T cells appear to be important in the control of viral replication (1, 2, 8–10). However, available data also suggest that EBV employs multiple mechanisms to evade the host immune response, and this may be critical to its ability to silently infect most of the population and establish latency in memory B cells (11, 12). For instance, EBV does not induce a strong type 1 interferon (IFN) response (13, 14); EBV also encodes an immunosuppressant interleukin-10 (IL-10) homologue within its genome (11, 12).

Here, we postulate that in some individuals, changes in the CD8 T-cell repertoire resulting from prior unrelated infections results in AIM immunopathology. Over the course of an individual’s life, encounters with various antigens leave imprints on the immune system that affect innate and adaptive immune responses in subsequent infections. These heterologous effects on immunity may be beneficial or harmful (15–22). Evidence for heterologous immunity mediated via T-cell cross-reactivity, even to unrelated viruses, and its impact on disease outcome in both mouse and human studies is continuing to increase (23–28). Mouse models show that T-cell cross-reactivity can change patterns of T-cell immunodominance (24), lead to the generation of narrowly focused T-cell repertoires and T-cell escape viral mutants (29), and sometimes confer a level of beneficial protective immunity impacting the difference between life and death (17, 30, 31) but at other times be detrimental, leading to more-severe disease with substantially altered pathology (17, 26, 32, 33).

While mechanistic studies of humans are difficult to perform, AIM provides a system amenable to mechanistic analyses. We and others have described heterologous immunity to three viruses that infect humans (influenza A virus [IAV], hepatitis C virus, and dengue virus) (34, 35) and more recently between dengue virus and Zika virus (36). IAV, the cause of influenza, is an important human pathogen, and “heterotypic” or protective heterologous immunity has been documented via both CD4 and CD8 T-cell cross-reactivity to IAV strains (37–39). *Mycobacterium bovis* BCG, live polio, and measles vaccines have been reported to decrease death due to unrelated pathogens in developing countries (40). Intriguingly, children vaccinated with BCG also had a 40% lower incidence of atopy (41). Detrimental heterologous immunity may help explain why young adults, who have complex, large, potentially cross-reactive memory T-cell pools, commonly get more severely ill with infections like those due to EBV, cytomegalovirus (CMV), varicella-zoster virus, and mumps virus, which normally are mild in younger children (42).

This study highlights the importance of virus-specific CD8 T-cell receptor (TCR) repertoires in the mediation of heterologous immunity. TCR sequence diversity is thought to enhance the surveillance efficiency of CD8 T cells and has been associated with improved viral control and reduced viral escape (34, 35, 37). We recently used TCR sequences to relate structural interactions between peptide-major histocompatibility complex (MHC) complexes and TCRs to the selection of TCR repertoires and the functional consequences of these interactions in response to the HLA-A2-restricted influenza virus A-M1_58_ (IAV-M1) epitope (43). CD8 TCR repertoires in response to common viruses (IAV, CMV) are highly diverse and individualized; this is often referred to as the “private specificity” of TCR repertoires. However, “public” clonotypes, which are defined by the use of the same V, J, or CDR3 amino acid sequences in many individuals, are favored for expansion, likely because of selection for optimal structural interactions (44–46). Our recent results (43) suggest that antigen-specific TCR repertoires have evolved “focused diversity,” i.e., public clonotypes with highly diverse private responses, to provide the ability to rapidly recognize their antigen, while retaining flexibility should the antigen mutate or to assist in rapid responses to a new cross-reactive pathogen. Although there are some recent limited reports of the TCR repertoire being linked to disease (47), the role specific TCRs play in the mediation of T-cell functional responses and disease outcome is still poorly understood.

Some AIM patients and healthy persistently EBV-infected donors have HLA-A2.01-restricted CD8 T-cell responses cross-reactive to an immunodominant and highly conserved IAV-encoded M1_58_ epitope (IAV-M1) and two EBV-encoded epitopes, EBV BMLF1_280_ (EBV-BM) and EBV-BLRF1_109_ (EBV-BR) (48, 49). We have also recently detected unique, functionally IAV-M1/EBV-BM cross-reactive oligoclonal CD8 TCR repertoires in five rare individuals who remain EBV seronegative (MA-EBV-SN) into their 4th decade of life, suggesting that cross-reactive CD8 T cells may protect from EBV infection (5). These studies provide a strong rationale for further examination of the potential role of both virus-specific and cross-reactive TCR repertoires in the mediation of clinical outcomes upon pathogen exposure. Mouse models of heterologous immunity have shown that the same epitope cross-reactive response can either be protective or induce immunopathology, depending on the individual’s history of infection and TCR repertoire (24, 49–54). Here we sought to determine if there is a correlation between the expansion of IAV-M1/EBV-BM cross-reactive T-cell responses and the severity of AIM during EBV infection. In addition, if these memory IAV-M1 cross-reactive responses had TCR repertoires different from those of seropositive persistently EBV-infected healthy donors (HD-SP) and if they differed between mild- and severe-AIM patients, this would be strong evidence that prior selection of cross-reactive IAV-M1 memory TCR repertoires influences the severity of AIM. Our hypothesis is that following infection of an immune host with a heterologous virus, cross-reactive T-cell responses, when present, are selectively expanded and impact the outcome of disease due to the heterologous virus. Thus, these studies have systematically examined whether CD8 T-cell cross-reactivity between an individual’s memory responses to IAV and EBV lytic antigens plays a role in the modification of antigen-specific CD8 population frequencies, function, and TCR repertoire and whether this correlates with disease outcome during AIM.

## RESULTS

### Characteristics of the study populations.

Over a 10-year period, we enrolled 32 AIM patients and 17 healthy persistently EBV-infected seropositive donors (HD-SP). In both groups, the median age was 20 years and there was equal representation of males and females (see Table S1 in the supplemental material). Because the goal of this study was to determine the role of cross-reactive CD8 T cells in the mediation of AIM severity and because the clinical presentation of AIM varied greatly between patients, we developed a strategy to score disease severity. Adenopathy, the most common sign of AIM on physical examination (our unpublished data), directly correlated (Fig. S3, graph i) with the degree of expansion of atypical lymphocytes, i.e., large granular lymphocytes documented on the peripheral blood smear and used as one of the diagnostic criteria for AIM. An inversion of the CD4/CD8 ratio in peripheral blood mononuclear cells (PBMCs) has long been associated with acute viral infection because of the large expansion of CD8 T cells, another pathognomic finding in AIM. We found that the CD4/CD8 ratio inversely correlated with the percentage of atypical lymphocytes (Fig. S3, graph ii). Thus, for some of the correlation studies, we used the percentage of atypical lymphocytes as a direct and quantitative measurement of AIM severity. However, in order to actually stratify each patient into the mild- or severe-AIM group, we scored AIM severity by using these same three direct measurements of AIM pathology, including the percentage of atypical lymphocytes in peripheral blood, the level of lymphadenopathy as scored by the same research nurse, and the CD4/CD8 ratio in PBMCs (details are in Materials and Methods) (Table S1). On the basis of this scoring strategy, AIM patients could be stratified into two groups, with severe-AIM patients having a significantly (Student *t* test) higher adenopathy score (6.5 ± 0.6 versus 3.3 ± 0.8; *n* = 15 or 16; *P* = 0.01) and percentage of atypical lymphocytes (44 ± 4.2 versus 23 ± 3.8; *n* = 15 or 16; *P* = 0.002) and a lower CD4/CD8 ratio (0.6 ± 0.08 versus 1.4 ± 0.1; *n* = 15 or 16; *P* = 0.001) than mild-AIM patients.

10.1128/mBio.01841-17.8 TABLE S1  Characteristics of the study population. Download TABLE S1, DOCX file, 0.1 MB. Copyright © 2017 Aslan et al. 2017 Aslan et al. This content is distributed under the terms of the Creative Commons Attribution 4.0 International license.

### Evidence of increased IAV/EBV CD8 T-cell cross-reactivity in severe-AIM patients by tetramer costaining directly *ex vivo* in PBMCs.

CD8 T-cell cross-reactivity can be complex and is easiest to demonstrate if costaining with two tetramers is successful, usually when the two epitopes have similar avidity to the same TCR (29, 48, 49). However, cross-reactive epitopes may not share sequence identity and weaker affinity for one of the ligands may, because of competition, reduce tetramer binding, as has been reported for MHC class I cross-reactive ligands in autoimmune diseases and tumor studies (55–57).

**FIG 1:**
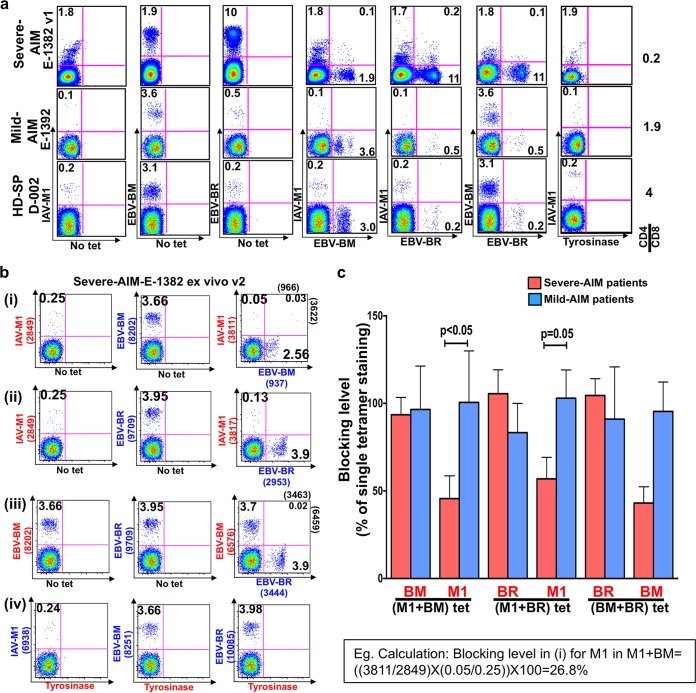
Evidence of increased IAV/EBV CD8 T-cell cross-reactivity in PBMCs of severe-AIM patients by tetramer costaining directly *ex vivo*. (a) One way to determine the frequency of cross-reactive CD8 T cells in peripheral blood sorted CD8 T cells is costaining of cells with different tetramer pairs as shown in this representative example, which shows that during the acute phase of AIM at visit 1 (v1), a severe-AIM donor (E-1382) has more IAV-M1+EBV-BM tetramer^+^ CD8 T cells than a mild-AIM donor (E-1392) and HD-SP (D-002). (b) This same severe-AIM patient at v2 showed a competitive reaction and mutual tetramer blocking of IAV-M1 and EBV-BM tetramer binding upon costaining with these two tetramers. Costaining with IAV-M1 and EBV-BR tetramers did not result in double-tetramer-positive cells, but the level of IAV-M1 tetramer^+^ cells declined compared to that of IAV-M1 single-tetramer-staining cells. There was no blockade upon costaining with EBV-BM and EBV-BR or IAV-M1 and control tyrosinase_369–377_ tetramers. In these studies, the exact same tetramers were used for the single-color control and double-tetramer staining. The values in parentheses are the MFIs of the populations indicated. (c) Significant IAV-M1-specific tetramer blockade by EBV-BM-specific tetramer and EBV-BR-specific tetramer was detected *ex vivo* in severe-AIM patients (*n* = 11) compared to mild-AIM patients (*n* = 7). On the *x* axis, the red text indicates the tetramer-specific response that was being blocked by the other tetramer. Below the line is the double-tetramer combination used for costaining. The estimated blocking level for each tetramer was calculated by the formula [(costained tetramer A MFI/alone tetramer A MFI) × (costained tetramer A%/alone tetramer A%)] × 100. The gating strategies used are shown in Fig. S1.

Costaining of CD8 T cells with epitope-specific tetramers is the most direct way to demonstrate cross-reactivity. Representative fluorescence-activated cell sorter (FACS) plots from patients in both severity groups demonstrate higher frequencies of circulating total IAV-M1 (without tetramer costaining), cross-reactive IAV-M1+EBV-BM, and cross-reactive IAV-M1+EBV-BR tetramer^+^ CD8 T cells in a representative severe-AIM patient than in a mild-AIM patient or a control HD-SP (Fig. 1a). These cross-reactive responses usually peaked at visit 1 or 2 and then declined at subsequent visits, along with the total EBV-BM- and EBV-BR-specific responses. In contrast, there was no evidence of cross-reactive responses to CMV pp65 by tetramer costaining during AIM in CMV-seropositive donors (data not shown). However, the tetramer costaining method has to be carefully controlled, as in some cases one tetramer binds with higher affinity and blocks the binding of the other (Fig. 1b and c) (49). This problem was particularly evident in the severe-AIM patient group, where there was significantly more blockade of the cross-reactive IAV-M1 tetramer binding by EBV-BM or EBV-BR tetramers *ex vivo* than staining in mild-AIM patients (Fig. 1b and c; Fig. S2a). T cells can also cross-react to EBV-BM and EBV-BR, and EBV-BM tetramer binding was also blocked by EBV-BR tetramer in some severe-AIM patients (Fig. 1c; Fig. S2a). Epitope-specific blockage of tetramer staining was also present in some AIM patient short-term cultures (Fig. S2b). Because of these issues with tetramer blocking affecting the accuracy of tetramer frequency determination, when we refer to IAV-M1, EBV-BM, or EBV-BR tetramer^+^ frequencies, we are referring to the total population by using data from the single tetramer staining frequencies (these frequencies will thus include the cross-reactive populations). Thus, ironically and very inconveniently, the blockade of the binding of one tetramer by the presence of a second tetramer is also further evidence of cross-reactivity.

10.1128/mBio.01841-17.1 FIG S1  (a) Representative *ex vivo* flow cytometry gating strategy. Sorted CD8^+^ T cells were gated with a lymphocyte gate, followed by forward scatter (FSC) A-FSC H (singlet) gating. Dead cells were excluded by live-dead violet viability kit labeling. CD3^+^ CD8^+^ T cells were derived from the live-cell gate. (b) Representative CD8 T-cell short-term culture flow cytometry gating strategy. Directly *ex vivo* isolated CD8 T cells were stimulated with chosen peptide-loaded T2 cells weekly for a minimum of 3 weeks. The lymphocyte gate was optimized to contain the larger actively proliferating T cells. These cells were further gated to detect live CD3^+^ CD8^+^ T cells. Download FIG S1, TIF file, 44.7 MB. Copyright © 2017 Aslan et al. 2017 Aslan et al. This content is distributed under the terms of the Creative Commons Attribution 4.0 International license.

10.1128/mBio.01841-17.2 FIG S2a  Representative examples of costaining with two tetramers simultaneously, resulting in blocking of tetramer binding when CD8 T-cell cross-reactivity is present directly *ex vivo*. (i) Costaining of freshly isolated CD8 T cells derived from a mild-AIM patient (E-1303) with M1 and BM tetramers showed 0.02% double tetramer^+^ cells. However, in the presence of BM tetramer, M1 tetramer^+^ cells declined to 0.06% compared to 0.25% in the presence of single M1 tetramer or the non-cross-reactive control tyrosinase-specific tetramer. A similar blockade of M1 tetramer was observed in the presence of BR tetramer costaining. There was also a blockade of the two EBV-specific tetramers, BM and BR. (ii) In a severe-AIM patient (E-1382), costaining of CD8 T cells directly *ex vivo* with M1 and BM tetramers showed a mutual blockade. M1 tetramer^+^ cells declined to 0.08% compared to 0.25% in the presence of M1 tetramer alone or a tyrosinase-specific tetramer. BM tetramer^+^ cells declined to 2.59% in the presence of M1 tetramer from 3.66% when BM tetramer was used alone. Also, in the presence of BR tetramer, the total M1 tetramer^+^ cell level declined to 0.13% compared to 0.24% in the presence of a tyrosinase-specific tetramer. There was no blockade between EBV-lytic epitope-specific tetramers. (iii) In a severe-AIM patient (E-1382) later during the acute phase of infection (visit 5), we observed different blocking patterns upon costaining with two tetramers compared to visit 2 staining, suggesting that the cross-reactive TcR repertoires were evolving over time. Red indicates blocked tetramers, and blue indicates blocking tetramers. Download FIG S2a, TIF file, 44.7 MB. Copyright © 2017 Aslan et al. 2017 Aslan et al. This content is distributed under the terms of the Creative Commons Attribution 4.0 International license.

10.1128/mBio.01841-17.3 FIG S2b  Representative examples of costaining with two tetramers simultaneously showing blocking of tetramer binding when CD8 T-cell cross-reactivity was present in short-term-cultured cells. (i) Culturing of CD8 T cells with BM peptide resulted in the proliferation of cross-reactive IAV-M1-specific cells (14%) in a severe-AIM patient (E-1325) at visit 8. However, upon costaining with M1- and BM-specific tetramers, the total BM tetramer^+^ cell percentage declined to 54% and the MFI dropped 11-fold compared to 60% with single BM tetramer or in the presence of tyrosinase-specific tetramer. There was no blocking of the cross-reactive M1 tetramer binding by BM tetramer. This indicates that the M1 tetramer was blocking BM tetramer binding on the cross-reactive cells. (ii) Culturing of CD8 T cells with M1 peptide promoted the growth of a smaller population of cross-reactive BM-specific cells. Costaining with M1 and BM tetramers did result in 0.16% double tetramer^+^ cells, and BM tetramer^+^ cells declined to a total of 0.66% compared to 1% with single BM tetramer or costaining with a tyrosinase-specific tetramer. (iii) In the BR-stimulated culture, there was an outgrowth of cross-reactive M1 cells with double M1^+^ BR^+^ tetramer^+^ cells at 2.3%. However, in the presence of BR costaining, cross-reactive M1 tetramer^+^ cells declined to 14.3% with a 16.5-fold decline in MFI compared to a frequency of 24% with single M1 tetramer or costaining with a tyrosinase-specific tetramer. These data indicate that BR tetramer blocked cross-reactive M1 tetramer binding. Red indicates blocked tetramers, and blue indicates blocking tetramers. Download FIG S2b, TIF file, 44.7 MB. Copyright © 2017 Aslan et al. 2017 Aslan et al. This content is distributed under the terms of the Creative Commons Attribution 4.0 International license.

10.1128/mBio.01841-17.4 FIG S3  The percentage of peripheral blood atypical T lymphocytes (*x* axis), which are diagnostic of EBV-induced AIM, directly correlated with the clinical severity score of lymphadenopathy (i) and inversely correlated with the peripheral blood CD4/CD8 ratio (ii). Download FIG S3, TIF file, 44.7 MB. Copyright © 2017 Aslan et al. 2017 Aslan et al. This content is distributed under the terms of the Creative Commons Attribution 4.0 International license.

### Directly *ex vivo* in PBMCs, only IAV-M1 and IAV-M1+EBV-BM tetramer^+^ CD8 T cells strongly correlate with AIM severity and predict an increased relative risk of severe AIM.

**FIG 2:**
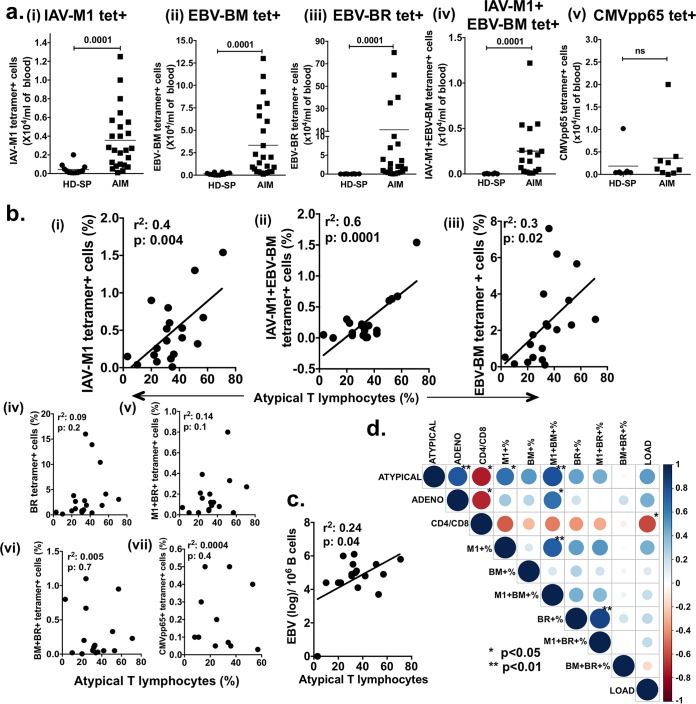
Only total IAV-M1 and IAV-M1+EBV-BM tetramer^+^ CD8 T cell levels in PBMCs analyzed *ex vivo* strongly correlate with AIM severity and predict the increased relative risk of severe AIM. (a) The average numbers of tetramer^+^ IAV-M1 (M1) (i), EBV-BM (BM) (ii) EBV-BR (BR) (iii) and IAV-M1+EBV-BM (M1^+^ BM^+^) (*n* = 23 to 25) (iv), but not CMV pp65-specific (*n* = 7 to 9) (v), CD8 T cells/ml of peripheral blood were significantly higher in AIM patients at the peak of their CD8 response than in healthy persistently infected EBV-seropositive donors (HD-SP) (*n* = 8 to 12) (Mann-Whitney U test). ns, not significant. (b) During the course of AIM, the peak (highest) frequency of tetramer^+^ IAV-M1 (i), IAV-M1+EBV-BM (ii), and EBV-BM (iii) CD8 T cells *ex vivo* directly correlated with the percentage of atypical lymphocytes; the peak frequency of tetramer^+^ EBV-BR (iv), IAV-M1+EBV-BR (v), EBV-BM+EBV-BR (vi), and CMV pp65 (vii) CD8 T cells *ex vivo* did not correlate with the percentage of atypical lymphocytes (*n* = 17 to 19 AIM patients per analysis). (c) During the course of EBV infection, the peak (greatest) viral load (measured as the genome copy number per B cell) had a weak direct correlation with disease severity measured as the percentage of atypical lymphocytes. (d) Display of pairwise correlations between variables of interest computed in a correlation matrix by using the Pearson correlation coefficient (the *P* values shown are adjusted for the number of multiple variant comparisons) and then graphically displayed as a matrix by using the corrplot R package with dark blue as the most positive correlation coefficient of 1 and dark red an inverse correlation coefficient of −1. Relative-risk analyses are shown in Table S2.

Significantly increased CD8 T-cell responses to total EBV-BM, to total EBV-BR, and to total IAV-M1 in AIM patients were detected directly *ex vivo* by tetramer staining (Fig. 2). Twenty-four-fold and 185-fold increases in the numbers of EBV-BM and EBV-BR tetramer^+^ cells/ml of blood, respectively, were observed, along with 10-fold and 25-fold increases in cross-reactive IAV-M1 and IAV-M1+EBV-BM tetramer^+^ cells/ml compared to those in HD-SP (Fig. 2a). This did not appear to be a nonspecific or bystander expansion of all memory cells, as an increase in the number of CMV pp65 tetramer^+^ cells/ml was not observed in CMV-seropositive AIM patients (Fig. 2a, graph v).

10.1128/mBio.01841-17.9 TABLE S2  Factors associated with an increased relative risk of a severe-AIM diagnosis. Download TABLE S2, DOCX file, 0.1 MB. Copyright © 2017 Aslan et al. 2017 Aslan et al. This content is distributed under the terms of the Creative Commons Attribution 4.0 International license.

We then questioned whether there was any evidence of a potential role for these cross-reactive IAV-M1 tetramer^+^ cells in the mediation of AIM severity. Interestingly, only some of these IAV and EBV epitope-specific CD8 T-cell responses correlated with disease severity. Only the *ex vivo* peak (highest) frequencies of total IAV-M1 and the cross-reactive double IAV-M1+EBV-BM tetramer^+^ subset, but not the IAV-M1+EBV-BR tetramer^+^ subset, directly correlated with the severity of AIM measured as the percentage of atypical lymphocytes (Fig. 2b). Interestingly, of the EBV-specific responses, only the total EBV-BM tetramer^+^ frequencies weakly correlated with AIM severity, but not the other early dominant epitope-specific response, EBV-BR or cross-reactive EBV-BM and EBV-BR tetramer^+^ cells. There was also no correlation between another memory population, CMV pp65 tetramer^+^ frequency and AIM severity (Fig. 2b). There was a weak correlation between AIM severity and the peak EBV load (Fig. 2c). It should also be noted that multivariate analysis of the peak total IAV-M1, EBV-BM, and EBV-BR tetramer^+^ frequencies *ex vivo* suggested that the total IAV-M1 tetramer^+^ frequencies were equivalently expanded within the mild- and severe-AIM groups during AIM to the EBV epitope responses, except in one case; the EBV-BR tetramer^+^ frequency was greater than the IAV-M1 tetramer frequency in the severe-AIM group (one-way analysis of variance [ANOVA] with Tukey’s multiple-comparison test, *P* < 0.04). However, the display of pairwise correlations between all of these variables of interest, when computed in a correlation matrix by using the Pearson correlation coefficient (Fig. 2d) and multivariate analysis, suggests that there is something unique about the total IAV-M1 tetramer^+^ response during AIM and in particular the double IAV-M1+EBV-BM tetramer^+^ cross-reactive response, which may drive AIM severity. Only these two factors significantly correlated with disease severity after adjustment for multivariate comparisons. It is possible, if not likely, that the majority of the total IAV-M1 tetramer^+^ cells in AIM were cross-reactive with EBV-BM, as their frequency directly correlated with the IAV-M1+EBV-BM tetramer^+^ frequency (Fig. 2d). In contrast, the frequency of cross-reactive IAV-M1+EBV-BR tetramer^+^ cells correlated with that of EBV-BR tetramer^+^ cells (Fig. 2d). In fact, relative-risk analyses also revealed that only total IAV-M1 tetramer^+^ (if ≥0.36%, relative risk = 4.9, odds ratio = 14, *P* < 0.05; Fisher’s exact test) and IAV-M1+EBV-BM tetramer^+^ (if ≥0.1%, relative risk = 5.8, odds ratio = 18.67, *P* < 0.02) peak frequencies (usually at visit 1 or 2) could predict an increased risk of developing severe AIM (Table S2). As in our highly controlled mouse studies of heterologous immunity (33, 50), these cross-reactive IAV-M1-specific CD8 T-cell responses that expanded during AIM were likely due to reactivated memory cells rather than *de novo* new naive responses, as all of these patients were IAV immune. This is further supported by the observation that, in naive cord blood, IAV-M1-specific CD8 T cells do not proliferate in response to EBV-BM or EBV-BR peptide stimulation (5). On the basis of these studies, we propose a link between IAV-M1 and EBV-BM cross-reactive T cells and AIM severity.

### Directly *ex vivo* in PBMCs, the mean frequency of total IAV-M1 and IAV-M1+EBV-BM tetramer^+^ CD8 T cells was increased in the severe-AIM group.

**FIG 3:**
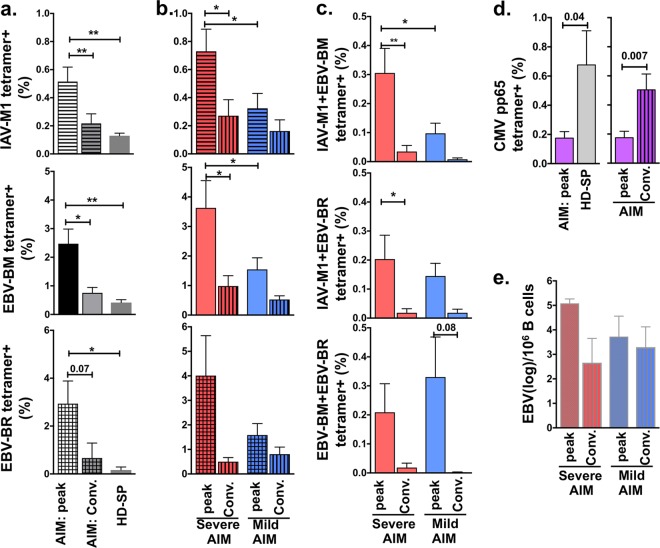
PBMCs in patients with severe AIM had a greater mean frequency of total IAV-M1, EBV-BM, and IAV-M1+EBV-BM tetramer^+^ CD8 T cells than mild-AIM patients (when analyzed *ex vivo*). (a) All AIM patients had a significantly higher mean peak frequency of total IAV-M1-, EBV-BM-, and EBV-BR-specific tetramer^+^ CD8 T cells than healthy persistently infected EBV-seropositive donors (HD-SP). (b) When patients were categorized into two groups on the basis of disease severity (see Materials and Methods), severe-AIM patients had a significantly higher mean peak frequency of total IAV-M1 and EBV-BM tetramer^+^ cells, but not EBV-BR, directly *ex vivo* in their PBMCs than mild-AIM patients (*n* = 8 to 22 donors per group). The *ex vivo* mean frequency of total IAV-M1 and EBV-BM tetramer^+^ cells significantly decreased from the peak to the convalescent (conv) phase in the severe-AIM group (*n* = 6 to 12 donors per group). (c) The mean peak frequency of IAV-M1+EBV-BM tetramer^+^ cells in severe-AIM patients was higher than that in mild-AIM patients but not that in the other two cross-reactive populations. (d) In CMV-seropositive donors, CMV pp65 tetramer^+^ CD8 T cells were lower in AIM patients than in HD-SP and higher in the convalescent phase than the peak CD8 T-cell responses during AIM (AIM patients, *n* = 11; convalescent [Conv.] AIM patients, *n* = 8; HD-SP, *n* = 11). (e) There was no significant difference in the mean peak or convalescent-phase EBV load (measured as the genome copy number [log] per 10^6^ B cells) between severe- and mild-AIM patients. The Student *t* test was used to compare two groups, and one-way ANOVA with Sidak’s multiple-comparison test was used to compare more than two. Severe-AIM groups, red; mild-AIM groups, blue. *, *P* < 0.05; **, *P* < 0.01.

Despite individual variation in EBV-specific responses, all AIM patients had significantly higher mean frequencies of circulating total IAV-M1 (5-fold), EBV-BM (5-fold), and EBV-BR (15-fold) tetramer^+^ cells, measured directly *ex vivo* in PBMCs than did HD-SP (Fig. 3a). Interestingly, as in the correlation studies, when the donors were segregated into mild- and severe-AIM groups, the mean frequencies of total IAV-M1 or EBV-BM tetramer^+^ cells were 2.3- and 2.4-fold significantly greater, respectively, in severe-AIM than in mild-AIM patients (Fig. 3b). Although the mean frequency of total EBV-BR tetramer^+^ cells was higher in the severe-AIM group, the difference did not reach statistical significance, mostly likely because of great variation in frequency, as some severe-AIM patients had a percentage of EBV-BR tetramer^+^ cells as low as 0.4%. Differences in frequencies between patient groups were not detected in the convalescent phase 6 to 12 months after the acute phase of AIM (Fig. 3b). This was most likely due to a significant 2.6- and 3.7-fold decrease in the mean frequency of IAV-M1 and EBV-BM tetramer^+^ cells, respectively, in severe-AIM patients from the acute to the convalescent phase of infection (Fig. 3b). In mild-AIM patients, the expansion of all three epitope-specific responses during the acute phase was so moderate that it did not differ from that in the convalescent phase.

There also was a selective significant 3-fold greater mean peak frequency of cross-reactive IAV-M1+EBV-BM tetramer^+^ cells in severe-AIM patients than in mild-AIM patients, while the frequencies of the other two cross-reactive populations, IAV-M1+EBV-BR and EBV-BM+EBV-BR tetramer^+^ cells, were similarly increased in both patient groups (Fig. 3c). As mentioned above, in young CMV-seropositive donors during AIM, there was no increase in CMV pp65 tetramer^+^ CD8 cells (Fig. 3d), which were generally not cross-reactive with EBV lytic epitopes, as assessed by double tetramer binding. In fact, CMV pp65 tetramer^+^ cells were diluted out by the EBV-specific response, resulting in a significantly lower frequency in the acute phase of AIM than in the convalescent phase or in HD-SP. As in the multivariate correlation analyses, when the donors were separated into mild- and severe-AIM groups, there was no significant difference in the mean EBV load measured as the genome copy number in B cells during the acute or convalescent phase (Fig. 3e). These results further support the proposed selective link between IAV-M1 and EBV-BM cross-reactive T cells and AIM severity. In subsequent studies, we examined whether the TCR repertoire and associated functional responses in this cross-reactive population could mechanistically account for the difference in disease severity between mild- and severe-AIM patients.

**FIG 4:**
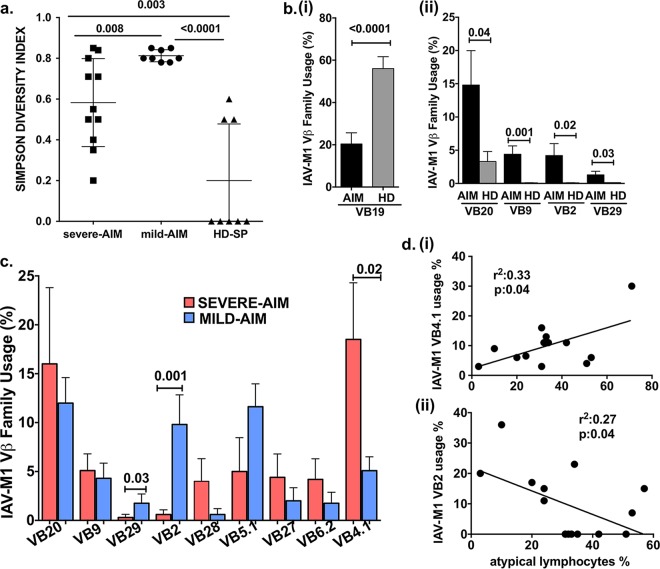
During AIM, patients with mild or severe disease have selected different TCR Vβ families in the expanding cross-reactive IAV-M1 tetramer^+^ cells (analyzed directly *ex vivo* in PBMCs). This suggests that each group had different cross-reactive TCR repertoires prior to acute EBV infection, with differential effects on disease severity. (a) Directly *ex vivo* sorted CD8 T cells from PBMCs were costained with IAV-M1 tetramer and Vβ-specific MAbs. Both severe-AIM (*n* = 11) and mild-AIM (*n* = 8) patients had more diverse IAV-M1-specific TCR Vβ family use than HD-SP (*n* = 8), as calculated by the SDI (see Materials and Methods). (b, part i) Decreased use of commonly used Vβ19 in the IAV-M1-specific TCR repertoire in AIM patients compared to that in HD-SP. (b, part ii) The Vβ repertoire of IAV-M1-specific T cells differed between AIM patients (*n* = 19) and HD-SP (*n* = 18), with increased use of Vβ20, -9, -2, and -29, which are more often associated with EBV-BM responses. (c) IAV-M1-specific TCR Vβ repertoires differed between mild- and severe-AIM patients. Severe-AIM patients (*n* = 11) preferentially used Vβ4.1, while mild-AIM patients (*n* = 8) preferentially used Vβ2 and Vβ29. (d, part i) Disease severity correlated directly with specific TCR Vβ use in AIM patients, suggesting that it plays a role in the mediation of disease severity. There was a direct correlation between the frequency of IAV-M1-specific TCR Vβ4.1 use and the peak frequency of atypical T lymphocytes during AIM. (d, part ii) There was an inverse correlation between the frequency of IAV-M1-specific TCR Vβ2 use and the peak frequency of atypical T lymphocytes. The TCR Vβ frequency of each donor is based on the mean of the first four visits (to enhance reproducibility), except for Vβ4.1, where it is based on the mean of all visits. The Student *t* test was used to compare two groups, and one-way ANOVA with Sidak’s multiple-comparison test was used to compare more than two groups.

Directly *ex vivo* in PBMCs, mild- and severe-AIM patients were shown to use different TCR Vβ repertoires in their total cross-reactive IAV-M1 tetramer^+^ cells, suggesting that the particular TCR repertoire played a role in disease severity, as these memory T cells were present in each donor prior to acute EBV infection. TCR repertoires of IAV-M1 tetramer^+^ cells in HD-SP and even young EBV-seronegative donors prior to infection with EBV are similar, with distinct characteristics. Studies have shown that they exhibit “focused diversity” in that they are highly diverse, differing between individuals, but strongly focused on TCR Vβ19 use and particular CDR3 motifs (5, 58). Therefore, if IAV-M1-specific TCR repertoires changed during AIM, this would be consistent with selective expansion of cross-reactive TCR repertoires rather than bystander activation of all IAV-M1 memory cells. Also, if the IAV-M1 TCR repertoire differed between mild- and severe-AIM patients, this would also suggest that they had different IAV-M1 TCR repertoires cross-reactive with EBV prior to being infected with EBV. This would be consistent with different IAV-M1 cross-reactive TCR repertoires driving different disease severities during AIM rather than EBV infection just activating IAV-M1 cross-reactive cells randomly. Therefore, we next questioned whether the IAV-M1-specific TCR repertoires of the three donor groups differed. This was determined by direct costaining with IAV-M1 tetramer and Vβ-specific monoclonal antibodies (MAbs) on *ex vivo* sorted CD8 T cells from fresh PBMCs. Using the Simpson diversity index (SDI) (59), we found that AIM patients (mean SDI, 0.7 ± 0.09; *n* = 19) had significantly more diverse IAV-M1-specific TCR Vβ repertoires than HD-SP (mean SDI, 0.2 ± 0.08; *n* = 8) (*P* < 0.0001, Student *t* test) (Fig. 4a). The TCR Vβ repertoire of IAV-M1-specific T cells differed between AIM patients and HD-SP. Consistent with previous reports (58, 60), TCR Vβ19, the most commonly used IAV-M1-specific TCR Vβ type in HD-SP (56.5% ± 5.6%, *n* = 18), significantly decreased in AIM patients (20.4% ± 5.2%, *n* = 18, *P* < 0.0001) (Fig. 4b, graph i). IAV-M1-specific TCR repertoires included multiple Vβ families, and Vβ use varied between HD-SP and AIM patients, as AIM patients preferentially used Vβ20, -9, -2, and -29 during the acute phase of infection (Fig. 4b, part ii). The IAV-M1-specific TCR Vβ repertoire also significantly differed between mild- and severe-AIM patients. Severe-AIM patients (*n* = 12) preferentially used many Vβ types, including Vβ20, -9, -28, -27, -6.2, and -4.1, but they only used Vβ4.1 significantly more than the mild-AIM patients. The mild-AIM patients (*n* = 8) also used multiple Vβ types but preferentially used Vβ29 and Vβ2 more than severe-AIM patients did during infection (Fig. 4c). Since severe- and mild-AIM patients had unique IAV-M1-specific TCR Vβ use, we questioned whether the use of a particular TCR Vβ type directly correlated with AIM severity. Indeed, there was a direct correlation between IAV-M1-specific TCR Vβ4.1 use and the peak frequency of atypical T lymphocytes, heralding severity of disease (Fig. 4d, part i). There was an inverse correlation between IAV-M1-specific TCR Vβ2 use and the peak frequency of atypical T lymphocytes (Fig. 4d, part ii). Thus, the cross-reactive IAV-M1 TCR repertoire differed between mild- and severe-AIM patients, and in fact, disease severity correlated with specific TCR Vβ use within the cross-reactive IAV-M1-specific cells in AIM patients. These results are strongly supportive of the concept that the cross-reactive IAV-M1 TCR repertoire that exists in an individual prior to EBV infection determines disease severity during AIM.

### Differences in the functionality of IAV/EBV cross-reactive CD8 T cells in different donor groups determined by combining tetramer staining and ICS in short-term *in vitro* culture.

**FIG 5:**
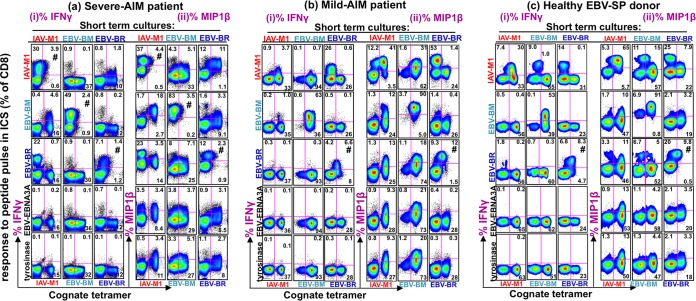
Representative examples of tetramer and intracellular cytokine costaining of CD8 T cells in short-term cultures suggesting that severe-AIM patients have the strongest functional cross-reactive responses. IAV-M1, EBV-BM, and EBV-BR peptide-stimulated short-term *in vitro* cultures generated from sorted CD8 T cells of representative severe-AIM (E-1302) (a) and mild-AIM (E-1392) (b) patients and an HD-SP (D-002) (c) were costained with cognate (same as the culture-stimulating peptide) tetramer and pulsed with cognate, cross-reactive, and control peptides (EBV latent epitope-specific peptide EBV-EBNA3A_509–604_ and self-peptide tyrosinase_369–377_). IFN-γ (i) and MIP-1β (ii) production was determined. The symbol # indicates that a cognate peptide pulse can result in such strong ligation of the TCR that it downregulates the TCR and thus the tetramer binding is hampered. (a) Representative severe-AIM patient who had more cross-reactive cytokine-producing cells (IFN-γ^+^ or MIP-1β^+^) in all three cultures. IAV-M1 tetramer^+^ cells (in IAV-M1 culture) produced both IFN-γ and MIP-1β in response to an EBV-BM peptide pulse, and EBV-BR tetramer^+^ cells (in EBV-BR culture) produced both IFN-γ and MIP-1β in response to an IAV-M1 peptide pulse. (b) In the mild-AIM patient, there was generally a much lower frequency of cross-reactive responses and only IAV-M1 tetramer^+^ cells produced both IFN-γ and MIP-1β in response to an EBV-BM peptide pulse. In the mild-AIM patient, the EBV-BR culture had a weaker type of cross-reactivity, as an IAV-M1 peptide pulse resulted in IFN-γ and MIP-1β production in cells that were not BR tetramer^+^. (c) The HD-SP had even lower frequencies of cross-reactive responses than the mild-AIM patient, and they were predominantly of the weaker type.

There is evidence that tetramer staining does not always correlate with functional responses to peptide (61). Recent studies suggest that tetramer staining may underestimate the frequencies of antigen-specific cells (43, 44). Therefore, it was important to also conduct functional studies, such as measurement of proliferation or cytokine production in response to stimulation with each ligand (33, 48, 49). Studies have also suggested that TCR use may be linked to particular T-cell functions (62). We have previously observed that the CD8 T-cell populations that expand *in vitro* in short-term cultures reflect the characteristics, both the TCR repertoire and the activation state, of the *in vivo* cells within a donor (5, 43, 48, 49, 60). We thus questioned whether the cross-reactive TCR repertoires under study were associated with qualitative differences in functional responses, such as proliferation or cytokine production upon interaction with either ligand *in vitro*. To study the qualitative differences and assess their functional profiles, we questioned whether these cross-reactive IAV-M1 tetramer^+^ cells would proliferate in IAV-M1-, EBV-BM-, and EBV-BR-stimulated short-term cultures and whether they produced either IFN-γ or MIP-1β in response to a peptide pulse. By combining cognate tetramer staining (tetramer specificity is the same as the stimulating peptide of the culture) with intracellular cytokine staining (ICS), we were able to determine if tetramer^+^ cells were making cytokines when pulsed with cross-reactive peptides (these are all other peptides except the peptide used to stimulate the culture, which can induce cytokines). Figure 5 shows examples of these combined tetramer-cytokine staining patterns of representative donors. Two types of cross-reactivity were observed, and the amount of each type of cross-reactivity present in any culture varied between donors. First, there could be a population of CD8 T cells with stronger, more obvious cross-reactivity where cognate tetramer^+^ cells made cytokines in response to a cross-reactive peptide pulse. The second population was evidence of weaker functional cross-reactivity, where CD8 T cells that had proliferated in response to the stimulating (cognate) peptide in culture did not bind the cognate tetramer but did make cytokines in response to cross-reactive peptide pulsing. We observed some differences between the representative donors from the three patient groups. The severe-AIM patient had the greatest frequency of cross-reactive IFN-γ-producing cells and the greatest number of different functionally cross-reactive populations between the EBV epitopes and IAV-M1 in all three cultures, resulting in IFN-γ production. This included the stronger cross-reactivity type, with four different cognate tetramer^+^ populations making IFN-γ upon a cross-reactive peptide pulse (IAV-M1 tet^+^/EBV-BM pulse, IAV-M1 tet^+^/EBV-BR pulse, EBV-BR tet^+^/IAV-M1 pulse, and EBV-BR tet^+^/EBV-BM pulse) (Fig. 5a). It also included the weaker cross-reactivity type with five different non-tetramer^+^ populations making IFN-γ in response to a cross-reactive peptide pulse (IAV-M1 culture/EBV-BR pulse, EBV-BM culture/IAV-M1 pulse, EBV-BM culture/EBV-BR pulse, EBV-BR culture/IAV-M1 pulse, and EBV-BR culture/EBV-BM pulse). The cross-reactive peptide pulses induced MIP-1β production even more often than IFN-γ (Fig. 5a), consistent with the fact that it is easier to induce MIP-1b with lower-avidity interactions (48). The representative mild-AIM patient and the HD-SP predominantly produced MIP-1β more than IFN-γ upon a cross-reactive peptide pulse and had more of the weaker type of cross-reactivity. This might suggest that the difference in the IAV-M1 memory cross-reactive TCR repertoire prior to EBV infection in the two AIM patient groups (Fig. 4) results in the expansion of stronger affinity highly functional cross-reactive responses in the severe-AIM group upon EBV infection than in the mild-AIM group. Examination of functional cross-reactivity in these same samples by directly gating on the cognate tetramer^+^ cells and showing an overlay of IFN-γ or MIP-1β histogram values for each peptide stimulation also demonstrated that the severe-AIM patient had a greater number of functionally cross-reactive responses between IAV-M1 and EBV-BM and -BR than the mild-AIM patient or HD-SP (Fig. S4). The histogram data also show that of the eight peptides tested, the cross-reactive responses were largely restricted to the IAV-M1, EBV-BM, and EBV-BR peptides, indicating high selectivity in this process. Overall, these representative data from each of the three patient groups suggest that there was greater proliferation of functionally cross-reactive cells, in particular IFN-γ-producing cells, in AIM patients than in HD-SP and more in severe-AIM patients than in mild-AIM patients. This conclusion is further supported by a statistical analysis of all of the subjects tested, as demonstrated below. In the next two sections, we summarize our systematic statistical analyses of the qualitative functional differences in both cell proliferation and cytokine production in the cross-reactive and cognate populations among all three donor groups that may help account for the differences in disease severity. However, using tetramer staining combined with ICS assays has certain drawbacks, as the peptide pulse in the ICS can lead to downmodulation of the TCR (e.g., Fig. 5a, IAV-M1 cognate tetramer with IAV-M1 pulse) and thus dramatically decrease tetramer binding and confound interpretation of the data. Therefore, to actually determine if there were significant functional differences in proliferation of the cross-reactive and cognate responses in the three patient groups in the different cell cultures, we examined tetramer frequency without peptide pulsing in the short-term *in vitro* cultures. To determine if there were significant functional differences in cytokine production of the cross-reactive and cognate responses in the three patient groups in the different cell cultures, we quantified the number of cells producing cytokines in response to cognate and a cross-reactive peptide pulse (without tetramer) and determined the frequencies of double-cytokine producers (IFN-γ^+^ MIP-1β^+^) and single-cytokine producers (MIP-1β^+^).

10.1128/mBio.01841-17.5 FIG S4  A severe-AIM patient had enhanced functionally cross-reactive CD8 T-cell responses in a short-term *in vitro* culture compared to those of a mild-AIM patient or HD-SP, as shown in histograms. IAV-M1-, EBV-BM-, and EBV-BR-specific short-term *in vitro* cultures generated from sorted CD8 T cells of representative severe-AIM (E-1302) (i) and mild-AIM (E-1392) (ii) patients and HD-SP (D002) (iii) were costained with cognate (same as the culture-stimulating peptide) tetramer and pulsed with cognate, cross-reactive, and control peptides; IFN-γ and MIP-1β production was determined. A cognate peptide pulse can result in such strong ligation of the TCR that it downregulates the TCR and thus tetramer binding is hampered. Examination of functional cross-reactivity in the same samples as in Fig. 5 by gating on the cognate tetramer^+^ cells in each culture and showing an overlay of IFN-γ or MIP-1β histogram values for each peptide pulse also demonstrates that the severe-AIM patient (i) had the greatest number of functional cross-reactive responses to IAV-M1 and EBV-BM or EBV-BR responses but not other peptides compared to those of a mild-AIM patient (ii) and an HD-SP (iii). Download FIG S4, TIF file, 25.1 MB. Copyright © 2017 Aslan et al. 2017 Aslan et al. This content is distributed under the terms of the Creative Commons Attribution 4.0 International license.

### Summary analyses of cross-reactive CD8 T-cell proliferation by tetramer frequency: unique profiles in donor groups with severe-AIM patients having the greatest proliferation of IAV/EBV cross-reactive CD8 T cells *in vitro*.

**FIG 6:**
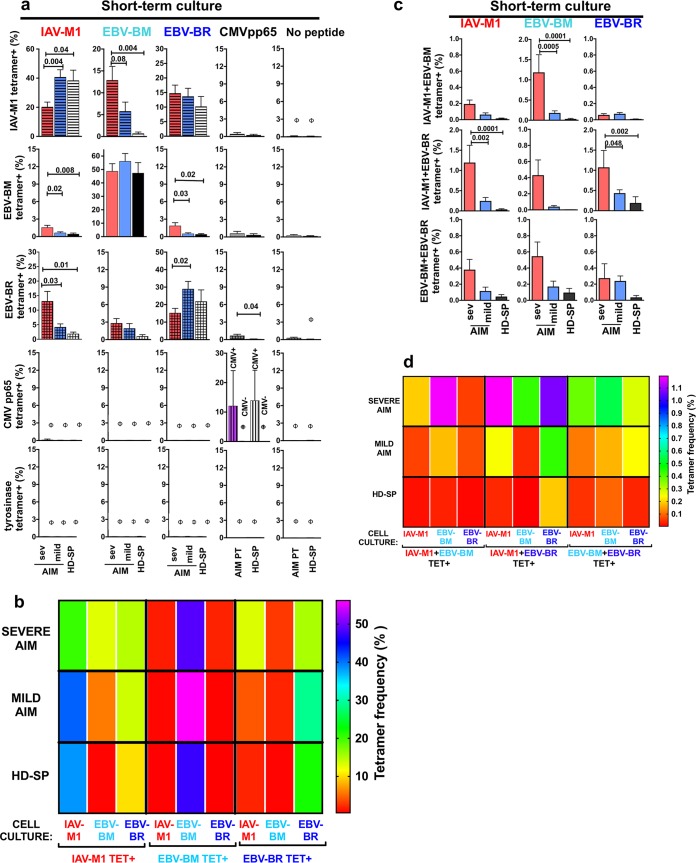
Severe-AIM patients have the greatest proliferation of IAV/EBV cross-reactive CD8 T cells in short-term *in vitro* cultures. In addition, each patient group maintains unique identifiable cognate and cross-reactive CD8 T-cell proliferation profiles upon peptide stimulation in short-term *in vitro* cultures, which are representative of their *in vivo* CD8 T-cell repertoires. The proliferation of IAV-M1-, EBV-BM-, and EBV-BR-specific cells in each culture was determined by costaining with pairs of tetramers (7 to 18 donors per group). In each culture, we determined the tetramer frequency of cognate (same as the culture) (a and b) and cross-reactive cells that were either single tetramer^+^ (a and b) or double tetramer^+^ (c and d). (a) Increased expansion of single tetramer^+^ cross-reactive cells in EBV-BM- and IAV-M1-stimulated cultures of cells from severe-AIM patients. There was significantly increased expansion of IAV-M1 tetramer^+^ cells in BM-stimulated cultures of cells from severe-AIM patients versus those from mild-AIM patients or HD-SP. Within the severe-AIM group, the cognate IAV-M1 tet^+^ frequency is lower than that in the mild-AIM group; instead, this group has a significantly increased frequency of cross-reactive IAV-M1 tet^+^ cells that grew in response to EBV-BM stimulation. Within the severe-AIM group, the cognate EBV-BR tet^+^ frequency is lower than that in mild-AIM patients; instead, this group has a significantly increased cross-reactive EBV-BR tet^+^ frequency in the IAV-M1-stimulated culture. Control cultures included short-term cultures with CMV pp65 or tyrosinase peptide or T2 cells (antigen-presenting cells) alone without a peptide, where the double tetramer frequencies were ≤0.1%, as indicated by the symbol φ. (b) Each donor group has unique profiles of cognate and cross-reactive IAV/EBV-specific CD8 T-cell proliferation that are highly significantly different from each other, as demonstrated in a heat map display with multivariate analyses. (c) Increased expansion of IAV/EBV double tetramer^+^ cross-reactive cells in severe (sev)-AIM patients. There were significantly higher frequencies of IAV-M1+EBV-BM tet^+^ cells in an EBV-BM-stimulated culture and IAV-M1+EBV-BR tet^+^ cells in both IAV-M1- and EBV-BR-stimulated cultures of cells from severe-AIM patients than in those of cells from mild-AIM patients or HD-SP. (The mean of all double tetramer^+^ frequencies pairing IAV-M1, EBV-BM, and EBV-BR tet^+^ with control tyrosinase tet^+^ was <0.1; the mean of all double tetramer^+^ frequencies in control CMV pp65-, tyrosinase-, and no-peptide-stimulated cultures was <0.1.) (d) Severe-AIM patients have a unique profile of double tetramer^+^ cross-reactive IAV/EBV-specific CD8 T-cell proliferation, as demonstrated in a heat map display with multivariate analyses. The Student *t* test was used to compare two groups, one-way ANOVA with Sidak’s multiple-comparison test was used to compare more than two groups, and multivariate analyses were done by two-way ANOVA with Tukey’s multiple-comparison test with adjustment for multiple comparisons. Details of the highly significant but complex multivariate statistical analyses for the heat maps (b and d) are shown in Fig. S5.

We examined if the proliferative capacity of the cognate (same peptide used to grow the culture) and cross-reactive CD8 T cells differed among the three different donor groups by determining the cognate and cross-reactive tetramer frequency profiles in IAV-M1, EBV-BM, and EBV-BR peptide-stimulated short-term cultures. The proliferation of IAV-M1-, EBV-BM-, and EBV-BR-specific cells in each culture was determined by costaining with pairs of tetramers (representative tetramer staining in cultures Fig. S2b). As previously reported (48), in the tetramer costaining studies, we observed two types of cross-reactive cells, those that costained with two tetramers and those that stained with only one tetramer but expanded *in vitro* in response to the cross-reactive peptide (also see Fig. S2b). In each culture, we determined the tetramer frequency of cognate (same as the culture) (Fig. 6a and b) and cross-reactive cells that were either single tetramer^+^ (Fig. 6a and b; Fig. S5a) or double tetramer^+^ (Fig. 6c and d; Fig. S5b). The cognate EBV-BM cells *in vitro* proliferated as well in AIM patients as in HD-SP, while cognate EBV-BR and IAV-M1 did not proliferate as well in severe-AIM patients as in HD-SP. Instead, the cross-reactive IAV-M1 responses dominated in culture, particularly in cells derived from severe-AIM patients. The cognate EBV-BR-specific cells may be in a more functionally exhausted state *in vivo*, thus proliferating poorly *in vitro* in severe-AIM patients. Each donor group had a unique profile of cognate and single cross-reactive IAV/EBV-specific CD8 T-cell proliferation, as demonstrated in the heat map display with multivariate analyses (Fig. 6b; Fig. S5).

10.1128/mBio.01841-17.6 FIG S5  Severe-AIM patients have the greatest expansion of IAV/EBV cross-reactive CD8 T cells in short-term *in vitro* cultures. In addition, each patient group maintains unique identifiable cognate and cross-reactive CD8 T-cell proliferation profiles upon peptide stimulation in short-term *in vitro* cultures, which are representative of their *in vivo* CD8 T-cell repertoires. The proliferation of IAV-M1-, EBV-BM-, and EBV-BR-specific cells in each culture was determined by costaining with pairs of tetramers (7 to 18 donors per group). In each culture, we determined the tetramer frequency of cognate (same as the culture) (a) and cross-reactive cells that were either single tetramer^+^ (a) or double tetramer^+^ (b). Each donor group has a unique profile of cognate and cross-reactive IAV/EBV-specific CD8 T-cell proliferation, as demonstrated in a heat map display with multivariate analyses. (a) Increased expansion of single tetramer^+^ cross-reactive cells in EBV-BM- and IAV-M1-stimulated cultures of cells from severe-AIM patients. There was significantly greater expansion of IAV-M1 tetramer^+^ (tet^+^) cells in EBV-BM-stimulated cultures of cells from severe-AIM patients than in cultures of cells from mild-AIM patients or HD-SP. Within the severe-AIM group, the cognate IAV-M1 tet^+^ frequency is lower than that in the mild-AIM group; instead, this group has a significantly increased frequency of cross-reactive IAV-M1 tet^+^ cells that grew in response to EBV-BM stimulation. Within the severe-AIM group, the cognate EBV-BR tet^+^ frequency is lower than that in cells from mild-AIM patients; instead, this group has a significantly increased cross-reactive EBV-BR tet^+^ frequency in the IAV-M1-stimulated culture. The symbol @ indicates that within the severe- and mild-AIM groups, the cognate EBV-BM tet^+^ frequency was significantly higher than that under all other conditions (*P* < 0.0001). The symbol & indicates that within the HD-SP group, the cognate EBV-BM tet^+^ frequency was significantly greater than under all other conditions, except that it was similar to that of cognate IAV-M1 tet^+^ cells (*P* < 0.0001). The symbol # indicates that within the mild-AIM group, cognate IAV-M1 tet^+^ frequency was significantly greater than under any other conditions, except that it was less than cognate EBV-BM tet^+^ cells (*P* < 0.0001). The symbol © indicates that within the HD-SP group, the cognate IAV-M1 tet^+^ frequency was significantly higher than under all other conditions, except that it was equal to that of cognate EBV-BM and EBV-BR tet^+^ cells (*P* < 0.0001). The symbol $ indicates that within the HD-SP and mild-AIM groups, the cognate EBV-BR tet^+^ frequency was significantly greater than that of all five cross-reactive populations (*P* < 0.05). (b) Increased expansion of IAV/EBV double tetramer^+^ cross-reactive cells in severe-AIM patients. There was a significantly higher frequency of IAV-M1+EBV-BM tet^+^ cells in EBV-BM-stimulated cultures and IAV-M1+EBV-BR tet^+^ cells in both IAV-M1- and EBV-BR-stimulated cultures of cells from severe-AIM patients than in cultures of cells from mild-AIM patients or HD-SP. (The mean of all double-tetramer frequencies pairing IAV-M1, EBV-BM, and EBV-BR tet^+^ with control tyrosinase tet^+^ was <0.1; the means of all double tetramers in control CMV pp65-, tyrosinase-, and no-peptide-stimulated cultures were <0.1.) The frequency of IAV-M1+EBV-BM tet^+^ cells was significantly higher upon EBV-BM stimulation than upon IAV-M1 or EBV-BR stimulation within the severe-AIM group. The frequencies of IAV-M1+EBV-BR tet^+^ cells were similar in either IAV-M1- and EBV-BR-stimulated cultures within the severe-AIM group. Multivariate analyses were done by two-way ANOVA with Tukey’s multiple-comparison test. *, *P* < 0.05; **, *P* < 0.01; ***, *P* < 0.001; ****, *P* < 0.0001 (*P* values were adjusted for multiple comparisons). Important significant and nonsignificant (N.S.) differences are shown on the graph to highlight the differences between patient groups. Download FIG S5, TIF file, 44.7 MB. Copyright © 2017 Aslan et al. 2017 Aslan et al. This content is distributed under the terms of the Creative Commons Attribution 4.0 International license.

There was also an increased expansion of IAV/EBV double tetramer^+^ cross-reactive cells in severe-AIM patients (Fig. 6c and d). There was a significantly higher frequency of three cross-reactive populations, IAV-M1+EBV-BM tet^+^ cells in BM-stimulated cultures and IAV-M1+EBV-BR tet^+^ cells in both IAV-M1- and EBV-BR-stimulated cultures, in severe-AIM patients than in mild-AIM patients or HD-SP. Severe-AIM patients had a unique specific profile of double tetramer^+^ cross-reactive IAV/EBV-specific CD8 T-cell proliferation, as demonstrated in a heat map display with multivariate analyses (Fig. 6d; Fig. S5b). Altogether, these results support the concept that in severe AIM there is a unique IAV-M1 TCR repertoire that is highly cross-reactive with EBV-BM, resulting in enhanced proliferation upon acute EBV infection.

### Summary analyses of cross-reactive CD8 T-cell cytokine production: severe-AIM patients have the greatest frequency of functional IFN-γ-producing cross-reactive cells *in vitro*.

To determine if there were significant functional differences in cytokine production of the cross-reactive responses in the three patient groups, we examined the short-term *in vitro* cultures by using peptide pulsing in an ICS and quantified double-cytokine producers (IFN-γ^+^ MIP-1β^+^) and single-cytokine producers (MIP-1β^+^).

**FIG 7:**
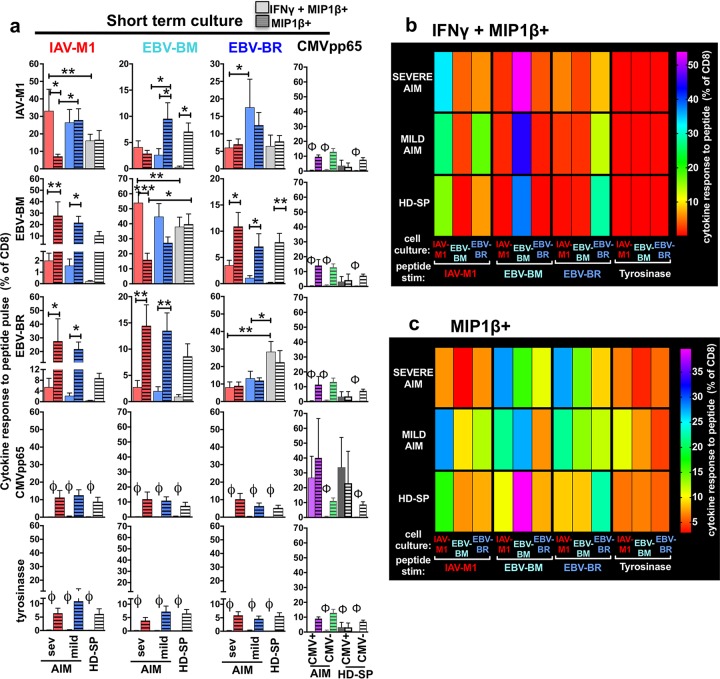
Cells from the severe-AIM, mild-AIM, and HD-SP groups each maintain a unique cognate and cross-reactive CD8 T-cell cytokine profile upon peptide stimulation in short-term *in vitro* cultures. Summary analyses of the cytokine profiles of IAV-M1, EBV-BM, and EBV-BR peptide-stimulated short-term cultures of cells from severe (sev)- and mild-AIM patients and HD-SP show significant differences in their functional responses to cognate (same as the culture peptide) and cross-reactive peptides (5 to 10 donors per group). The cytokine production of IAV-M1-, EBV-BM-, and EBV-BR-specific cells in each culture was determined by stimulation with each of the peptides in an intracellular cytokine assay and determination of the frequency of either double-cytokine-producing (IFNγ + MIP1β+) cells (a and b) or MIP-1β-only-producing (MIP1β+) cells (a and c). (a) Greater expansion of functional IFN-γ^+^ cross-reactive responses in severe-AIM patients versus mild-AIM patients in IAV-M1- and EBV-BM-stimulated rather than EBV-BR-stimulated short-term cultures. There was a significantly higher frequency of cognate IAV-M1 or cognate EBV-BM IFN-γ versus MIP-1β-only-producing cells in severe-AIM patients than in mild-AIM patients or HD-SP. There was a significantly lower frequency of cognate EBV-BR IFN-γ^+^ cells in severe-AIM and mild-AIM patients than in HD-SP. In both patient groups, cross-reactive peptides induce significantly more MIP-1β than IFN-γ, except upon an IAV-M1 pulse of either EBV-BM or EBV-BR cultures from severe-AIM patients. Also, an IAV-M1 pulse of EBV-BR cultures resulted in greater IFN-γ production by mild-AIM patients than by severe-AIM patients. Control cultures included short-term cultures with CMV pp65 or tyrosinase peptide (not shown), where the frequencies of IFN-γ^+^ cells were ≤0.1%, as indicated by the symbol φ. *, *P* < 0.05; **, *P* < 0.01. (b) Each donor group has a unique profile of cognate and cross-reactive IAV/EBV-specific IFN-γ^+^ CD8 T cells, as demonstrated in a heat map display with multivariate analyses. A cognate EBV-BM peptide pulse induced the greatest frequency of IFN-γ^+^ cells in all donors groups. In severe- and mild-AIM patients, it was significantly higher than all other populations, including cognate EBV-BR and IAV-M1, while in HD-SP, there were equal frequencies of cognate EBV-BR IFN-γ^+^ cells. A cognate IAV-M1 pulse in all donor groups induced the next greatest frequency of IFN-γ^+^ cells. A cognate EBV-BR peptide pulse induced the greatest frequency of IFN-γ^+^ in HD-SP. However, in both severe- and mild-AIM patients, the cognate EBV-BR IFN-γ^+^ cell level was so low that it was not significantly different from that of any other condition, including tyrosinase peptide pulse controls, except that it was significantly less than cognates EBV-BM and IAV-M1 in severe AIM and only cognate EBV-BM in mild AIM. (c) Each donor group has a unique profile of cognate and cross-reactive IAV/EBV-specific MIP-1β^+^ CD8 T cells, as demonstrated in a heat map display with multivariate analyses. In severe-AIM patients, a cross-reactive EBV-BM and EBV-BR peptide pulse of IAV-M1 cultures induces the greatest frequency of MIP-1β^+^ cells, significantly greater than cognate IAV-M1 and greater than their counterparts in HD-SP. In mild-AIM patients, the cognate IAV-M1 and EBV-BM peptide pulse induced the greatest number of MIP-1β^+^ cells. In HD-SP, the cognate EBV-BM peptide pulse induced the greatest number of MIP-1β^+^ cells in comparison to all other conditions and significantly more than cognate EBV-BM in severe-AIM patients. The Student *t* test was used to compare two groups, one-way ANOVA with Sidak’s multiple-comparison test was used to compare more than two groups, and multivariate analyses were performed by two-way ANOVA with Tukey’s multiple-comparison test. *, *P* < 0.05; **, *P* < 0.01; ***, *P* < 0.001 (*P* values are adjusted for multiple comparisons). All of the multiple important significant and nonsignificant differences in these graphs are shown in Fig. S6 to highlight differences between the patient groups.

Cross-reactive CD8 T cells proliferated and produced cytokines in response to cognate and cross-reactive peptide pulses with unique functional patterns in each patient group. In particular, the ratio of double-cytokine (IFN-γ^+^ MIP-1β^+^) to single-cytokine (MIP-1β^+^) producers differed between the groups. In the severe-AIM group, both the IAV-M1 and EBV-BM short-term cultures had significantly more double-cytokine-producing (IFN-γ^+^ MIP-1β^+^) cells than single-cytokine-producing (MIP-1β^+^) cells in response to the cognate peptide pulse than the mild-AIM and HD-SP groups (Fig. 7a). This suggests that severe-AIM patients have more EBV-BM- and IAV-M1-responding cells *in vivo* that have differentiated into IFN-γ-producing cells than mild-AIM patients or HD-SP. In contrast, in the severe- and mild-AIM groups, the EBV-BR short-term cultures had significantly fewer IFN-γ-producing cells in response to EBV-BR peptide than in the HD-SP group (Fig. 7a), further suggesting that these EBV-BR-specific cells might be partially functionally exhausted. Each donor group has a highly unique profile of cognate and cross-reactive IAV/EBV-specific IFN-γ^+^ (Fig. 7b) and/or MIP1β^+^ (Fig. 7c) CD8 T cells, as demonstrated in a heat map display with multivariate analyses (Fig. S6).

10.1128/mBio.01841-17.7 FIG S6  Severe-AIM, mild-AIM, and HD-SP groups each maintain a unique cognate and cross-reactive CD8 T-cell cytokine profile upon peptide stimulation in short-term cultures. The heat map display with multivariate analyses of the cytokine profiles of IAV-M1, EBV-BM, and EBV-BR peptide-stimulated short-term cultures from severe (sev)- and mild-AIM patients and HD-SP shows significant differences in their functional responses to cognate (same as the culture peptide) and cross-reactive peptides (5 to 10 donors per group). The cytokine production of IAV-M1-, EBV-BM-, and EBV-BR-specific cells was determined for each culture by stimulation with each of the peptides in an intracellular cytokine assay and determination of the frequency of either double-cytokine-producing (IFNγ + MIP1β+) cells (a) or MIP-1β-only-producing (MIP1β+) cells (b). (a) Greater expansion of functional IFN-γ^+^ cross-reactive responses in severe-AIM than in mild-AIM patients in IAV-M1- and EBV-BM-stimulated rather than EBV-BR-stimulated short-term cultures. There was a significantly greater frequency of cognate IAV-M1 or cognate EBV-BM IFN-γ versus MIP-1β-only-producing cells in severe-AIM patients than in mild-AIM patients or HD-SP. There was a significantly lower frequency of cognate EBV-BR IFN-γ^+^ cells in severe-AIM and mild-AIM patients than in HD-SP. In both patient groups, cross-reactive peptides induced significantly more MIP-1β than IFN-γ, except upon an IAV-M1 pulse of either an EBV-BM or an EBV-BR culture of cells from severe-AIM patients. Also, in mild-AIM patients, an IAV-M1 pulse of an EBV-BR culture resulted in greater IFN-γ production by mild-AIM patients than by severe-AIM patients. A cognate EBV-BM peptide pulse induced the greatest frequency of IFN-γ^+^ cells in all donors groups; in the severe- and mild-AIM groups, it was significantly higher than in all other populations, including cognate EBV-BR and IAV-M1 (the symbol @ indicates that the cognate EBV-BM IFN-γ^+^ frequency was greater than under all other conditions in severe- and mild-AIM patients, *P* < 0.0001), while in HD-SP, there were equal frequencies of cognate EBV-BR IFN-γ^+^ cells (the symbol & indicates that the cognate EBV-BM IFN-γ^+^ frequency was significantly greater than under all other conditions, except cognate EBV-BR in the HD-SP group, *P* < 0.0001). A cognate IAV-M1 pulse in all donor groups induced the next greatest frequency of IFN-γ^+^ cells (the symbol # indicates that the cognate IAV-M1 IFN-γ^+^ frequency was significantly higher than under all other conditions, including cognate EBV-BR, in severe-AIM donors, except that it was less than that of cognate EBV-BM [*P* < 0.01, *P* < 0.001]; the symbol © indicates that the cognate IAV-M1 IFN-γ^+^ frequency was significantly higher than under all other conditions in mild-AIM patients [*P* < 0.0001] and HD-SP [*P* < 0.05], except that it was similar to that obtained with a cognate EBV-BR and cross-reactive IAV-M1 peptide pulse of an EBV-BR culture and less than that obtained with cognate EBV-BM). A cognate EBV-BR peptide pulse induced the highest frequency of IFN-γ^+^ in HD-SP (the symbol $ indicates that in HD-SP, the cognate EBV-BR IFN-γ^+^ frequency was significantly higher than under all other conditions [*P* < 0.001] and it was not significantly different from cognates EBV-BM and IAV-M1). However, in both severe- and mild-AIM patients, cognate EBV-BR IFN-γ^+^ cell levels were so low that that they were not significantly different from those found under any other condition, including tyrosine peptide pulse controls, except that they were significantly lower than those obtained with cognates EBV-BM and IAV-M1 in severe-AIM patients and only cognate EBV-BM in mild-AIM patients. (b) Each donor group has a unique profile of cognate and cross-reactive IAV/EBV-specific MIP-1β^+^ CD8 T cells, as demonstrated in a heat map display with multivariate analyses. In the severe-AIM patients, a cross-reactive EBV-BM and EBV-BR peptide pulse of IAV-M1 cultures induces the greatest frequency of MIP-1β^+^ cells, significantly greater than cognate IAV-M1 and greater than their counterparts in HD-SP. In mild-AIM patients the cognate IAV-M1 and EBV-BM peptide pulse induced the greatest number of MIP-1β^+^ cells. In HD-SP, the cognate EBV-BM peptide pulse induced the greatest number of MIP-1β^+^ cells in comparison to all other conditions and significantly more than cognate EBV-BM in severe-AIM patients. Multivariate analyses were performed with two-way ANOVA with Tukey’s multiple-comparison test. *, *P* < 0.05; **, *P* < 0.01; ***, *P* < 0.001; ****, *P* < 0.0001 (*P* values are adjusted for multiple comparisons). Important significant and nonsignificant (ns) differences are shown on the graph to highlight difference between the patient groups. Download FIG S6, TIF file, 44.7 MB. Copyright © 2017 Aslan et al. 2017 Aslan et al. This content is distributed under the terms of the Creative Commons Attribution 4.0 International license.

Altogether, these functional studies demonstrate that each donor group had unique functional characteristics in each cognate and cross-reactive response, suggesting that these responses may have been triggered differently by the same ligands *in vivo*; one way that this could occur is if the TCR repertoires for EBV and IAV epitopes differed in these donor groups. Our TCR repertoire data (Fig. 4) suggest that this is the case for the cross-reactive IAV-M1 TCR repertoire. The cross-reactive memory IAV-M1 TCR repertoire that an individual has available to be activated early immediately upon EBV infection long before the new naive EBV epitope-specific response fully arises differs between donors (43, 53) and may well determine the functional profile of these cells upon exposure to EBV antigens. Thus, the greater expansion of functional IFN-γ-producing cross-reactive responses in severe-AIM patients versus mild-AIM patients in IAV-M1- and EBV-BM-stimulated, but not EBV-BR-stimulated, short-term cultures demonstrates a strong functional cross-reactivity to EBV-BM and IAV-MI and is consistent with the idea that this particular cross-reactivity plays a role in the mediation of AIM severity.

## DISCUSSION

In these systematic studies, multiple complementary methods demonstrated that selective CD8 cross-reactive TCR repertoires (Fig. 4) between an individual’s memory responses to IAV-M1 and the early antigen EBV-BM played a role in the modification of antigen-specific CD8 T-cell frequencies and functions and disease severity during the acute phase of EBV infection. Increases in the frequencies and absolute total numbers of IAV-M1, EBV-BM, IAV-M1+EBV-BM, and EBV-BR but not CMV pp65 tetramer^+^ cells were observed during AIM. However, total IAV-M1 and IAV-M1+EBV-BM cross-reactive CD8 T-cell frequencies were the only tetramer^+^ responses *ex vivo* in PBMCs that strongly directly correlated with AIM severity and that were predictive of severe disease by relative-risk analysis (Fig. 1, 2). The fact that total IAV-M1 and IAV-M1+EBV-BM cross-reactive CD8 T-cell frequencies correlated with each other suggests that the majority of the IAV-M1 tetramer^+^ cells present during AIM may be cross-reactive with EBV-BM (Fig. 2). When AIM patients were stratified into groups based on AIM severity, i.e., the mean peak frequencies in donors visiting the clinic at different times, only total IAV-M1, IAV-M1+EBV-BM and total EBV-BM tetramer^+^ CD8 T-cell counts were significantly higher in severe versus mild cases of AIM. In contrast, the mean peak frequencies of the other early EBV antigen, total EBV-BR, or cross-reactive IAV-M1+EBV-BR and EBV-BM+EBV-BR or CMV pp65 tetramer^+^ CD8 T cells did not differ between mild- and severe-AIM patients (Fig. 3). The fact that these cross-reactive memory IAV-M1 tetramer^+^ cells, which were already present at a high frequency in the donor prior to EBV infection ready to be activated immediately upon virus exposure, had unique TCR repertoires depending on whether the donor had mild or severe disease strongly suggests that these cells play a role in the mediation of disease severity. We show that there were higher numbers of IAV-M1 tetramer^+^ CD8 T cells cross-reactive with EBV-BM not only directly *ex vivo* in PBMCs (Fig. 1 to 3) but also in short-term cultures (Fig. 5 to 7). The short-term cultures further helped demonstrate that the three donor groups (severe AIM, mild AIM, and HD-SP) also had multiple qualitatively significantly different functional responses to EBV-BM and EBV-BR, as well as to cross-reactive IAV-M1 ligand. For instance, in severe-AIM versus mild-AIM patients, there was a greater proliferation of functional IFN-γ-producing (31, 33, 63) cross-reactive cells in IAV-M1 and EBV-BM than in EBV-BR-stimulated cultures (Fig. 5 to 7). Altogether, these results support the concept that cross-reactive CD8 memory T-cell responses with unique TCR repertoires and altered functional capacity play a role in determining disease severity during EBV infection and contribute to the induction of AIM.

By far, the strongest evidence that the cross-reactive IAV-M1 memory responses determine disease severity comes from an examination of the TCR repertoire use of this population in mild- and severe-AIM patients. This study uniquely correlates the severity of a human viral disease, AIM, with T-cell repertoire use, in this case, the cross-reactive IAV-M1-specific TCR repertoire. The IAV-M1 memory TCR repertoire during AIM was completely different from that in HD-SP, as it is being driven to expand in response to cross-reactive ligands on EBV rather than its original ligand IAV-M1, most likely predominantly by EBV-BM. The cross-reactive IAV-M1 repertoire in AIM patients was much more polyclonal than in HD-SP, and it used Vβ types that are commonly associated with EBV-BM, such as Vβ20, -9, -2, and -29 (44, 60), instead of the classic Vβ19 associated with IAV-M1 in HD-SP (Fig. 4) (5, 43, 64). What is most striking is that mild-AIM patients used different Vβ families, such as Vβ2 and -29, in their IAV-M1 response, and in fact, there was an inverse correlation between Vβ2 use and AIM severity. Since each individual has a different memory TCR repertoire in response to IAV-M1 at the clonal level, a phenomenon known as private specificity, each person has a different IAV-specific memory cross-reactive TCR repertoire with distinct subsets that may respond to EBV-BM. Our results suggest that those individuals who had more IAV-M1 Vβ2 clones cross-reactive to EBV-BM were more likely to have the mildest form of AIM. Those individuals who had the most IAV-M1 Vβ4.1 memory clones cross-reactive to EBV-BM had the most severe form of AIM, with a direct correlation between the frequency of Vβ4.1 use and AIM severity. This is certainly not consistent with a random activation of all IAV-M1 memory populations equally in both groups, such as that which might be expected if the viral load and virus-induced cytokines just drove the activation of all memory cells or even all cross-reactive memory cells. These results strongly suggest that the particular cross-reactive memory IAV-M1 TCR repertoire each individual had before being infected with EBV determined whether the patients developed mild or severe AIM. We have previously demonstrated that if responses are of lower avidity, as in this case, where the peptide sequences are fairly dissimilar, then this is more likely to activate a low expansion of a polyclonal population rather than a highly expanding narrow repertoire, which occurs in high-affinity often more protective cross-reactive responses (29, 60). For instance, we have observed that there is a unique high-avidity oligoclonal TCR repertoire in IAV-M1 memory cells cross-reactive with EBV-BM in the rare 5% of middle-aged individuals who never become infected with EBV (5). Disease etiology and diagnosis by TCR repertoire analysis are beginning to gain more attention as technology improves (47). Differences in virus-specific versus cross-reactive alphaherpesvirus-specific CD8 TCR repertoires have been described (65), and TCR repertoires are being linked to disease (47). For instance, using high-throughput sequencing of patients with MS, a disease associated with AIM, the TCR repertoire of cerebrospinal fluid was found to be distinct from that of blood and enriched in EBV-reactive CD8 T cells (66). Newly defined analytic tools, TCRdist and TCRdiv (repertoire distance and diversity measurements) (44), and the GLIPH algorithm (45) have been developed and may be very useful in identifying potential ligands that have been difficult to identify by using public features of the TCR repertoire that are common to donors with a particular disease like mycobacterial infection, tumors, or autoimmunity.

How, then, can different TCR repertoires influence disease outcome? Our findings are consistent with TCR activation to function differently by cross-reactive and cognate ligands. Previous studies (48, 49, 67) have shown that the TCR/CD3 complex is one of the most sophisticated immunologic signaling molecules and is capable of scalable signaling by recruiting at least 10 different immunoreceptor tyrosine-based activation motifs that control different signaling pathways (68, 69). The CD8 molecule has also been shown to be important for modulation of the TCR-MHC peptide interaction and subsequent signaling. CD8 has been shown to be particularly crucial in the activation of cross-reactive responses (70, 71). In severe-AIM patients, there was much stronger IFN-γ production by IAV-M1- and EBV-BM-specific cells than by cells with other specificities (Fig. 5 and 6). In contrast, the EBV-BR response appeared to be partially exhausted, as these T cells proliferated less and made less IFN-γ than those from HD-SP (Fig. 5 and 6). This is consistent with data that demonstrate that there is differential expression of PD1, an exhaustion marker, based on the specific TCR use of EBV-BR-specific cells (72). Perhaps the equally prevalent cross-reactive IAV-M1+EBV-BR tetramer^+^ cells in severe-AIM EBV-BR cultures have lower avidity for EBV-BR than non-cross-reactive EBV-BR-specific cells do and are thus less likely to be exhausted. In mouse models of heterologous immunity, IFN-γ has been a major player in the mediation of severe immunopathology in the lung and in fat (17, 26, 33). We have previously reported that T-cell exhaustion is a mechanism that successfully prevents severe morbidity and death due to a large dose of an overly aggressive persistent virus, lymphocytic choriomeningitis virus (LCMV) clone 13 (73). Perhaps the fact that a large part of the EBV-BR response becomes exhausted explains why it may not directly correlate with the severity of immunopathology.

These studies also provide further evidence that tetramer and cytokine frequency measurements are two different methods to determine the sizes of antigen-specific responses and that, depending on the patient group and the antigen, they do not always correlate. For instance, in the IAV-M1 culture of the HD-SP group, there was a 3-fold higher frequency of IAV-M1 tetramer^+^ cells (50%) (Fig. 6a) than double-cytokine producers in response to an IAV-M1 pulse (15%) (Fig. 7a). In contrast, in the cross-reactive responses of the severe-AIM group in the EBV-BM culture, there was an 11-fold greater frequency of cells producing MIP-1β in response to an IAV-M1 pulse (18% with the background subtracted) (Fig. 7a) than in the parallel IAV-M1 tetramer staining (1.6%) (Fig. 6a). This is consistent with more recent TCR sequencing data showing that tetramers may not always bind all of the predicted antigen-specific T cells (44, 45).

Hypothetically, the presence of cross-reactive IAV-M1 memory cells may alter the normal balance between EBV immune evasion strategies that normally would lead to delayed activation of CD8 responses resulting in silent persistent infection of B cells. High frequencies of cross-reactive IAV-M1 resident memory CD8 T cells in the tonsils of AIM patients may become activated early in EBV infection and may be the only CD8 T-cell response available for 3 to 4 weeks, before new naive EBV-specific CD8 T cells are activated. Unfortunately, thus far, it has not been possible to study this 4-week window between EBV exposure and the development of AIM. Perhaps their presence prevents EBV from immunosuppressing the tonsillar environment, where the virus normally moves silently from the tonsillar epithelium into B cells. This is thought to occur normally in the majority of individuals infected asymptomatically with EBV. EBV induces host IL-10, encodes a viral IL-10 homologue, and is a weak inducer of type 1 IFN (3, 12, 74). The coincident presence of activated cross-reactive IAV-M1 memory cells and EBV-induced immunosuppression may lead to dysregulated immune responses by way of early IFN-γ responses that occur during that 4-week window after EBV exposure and before the development of new naive EBV-specific CD8 T-cell responses and AIM, which leads to their overactivation.

Most likely there are other factors that may contribute to AIM induction and severity. For instance, in mild-AIM patients, there could be cross-reactive TCR repertoires that are beneficial, as suggested by the inverse correlation of some Vβ types with disease severity (Fig. 4). There are some hints in these studies that perhaps IAV-M1 and EBV-BR cross-reactivity may be beneficial to some patients and actually help keep disease severity milder. Also, there is a weak correlation with the viral load and AIM severity and a significantly increased relative risk if the viral load is extremely heavy (>4.5 log_10_). A heavier viral load for a protracted period of time could contribute to driving these altered immune responses in all AIM patients. However, unlike the cross-reactive IAV-M1 tetramer^+^ frequency, this factor is not consistent in all mild-AIM patients, as some mild-AIM patients have extremely heavy viral loads.

Unfortunately, studies of humans are not amenable to direct cause-and-effect testing of our hypothesis that cross-reactive CD8 memory T cells can mediate disease severity during viral infections. However, we have directly tested these theories in our mouse models multiple times by using many techniques, including cross-reactive memory CD8 T-cell adoptive transfer (49, 51). Thus far, in mouse studies, we have never found the viral load to correlate directly with the severity of pathology (50). In fact, in LCMV-immune mice infected with vaccinia virus, the viral load is almost always lighter than that in naive controls (17, 31, 49–51). However, depending on the private specificity of the cross-reactive memory CD8 TCR repertoire, some mice develop severe fatty necrosis of their abdominal fat. Adoptive-transfer experiments showed that all three recipients of one immune donor would have the same outcome whether it was no pathology or different levels of fatty necrosis. CD8 depletion studies and anti-IFN-γ antibody treatment demonstrated that both of these factors were important in the mediation of the severity of this pathology. Our present findings are highly similar to those of our studies with IAV-immune mice challenged intranasally with LCMV, where the mice develop very severe lung pathology similar to that seen in the 1918 influenza pandemic (33). In that model, there is an increased viral load and low-affinity, low-frequency memory CD8 T cells cross-reactive to LCMV and IAV that directly mediate the severe lung pathology via excess IFN-γ. These cross-reactive responses were at such low frequency that the causal relationship needed to be shown by correlating their preinfection frequency with the subsequent severity of lung pathology, by blocking by the peptide tolerization technique, or by preventing pathology by immunizing mice with IAV mutants lacking the cross-reactive epitopes (33). Pathology could also be prevented by using anti-IFN-γ antibody. These studies suggest that early activation of low-affinity cross-reactive cells that do not expand dramatically, such as was seen here in AIM, can completely alter the immune response to the new pathogen, leading to immunopathology. In contrast, generally dominant high-affinity cross-reactive responses are more likely to lead to more oligoclonal TCR repertoires that are more protective, as we have seen in middle-aged EBV-seronegative donors (5, 24, 29).

These results suggest that an individual’s history of infection, in particular, the individual’s memory TCR repertoire, may help to explain variations in human disease thought previously to be only due to genetic differences, the physiological condition of the patient, or the inoculation route and dose used (17, 22, 26, 31). Additional studies are needed to clarify instances and mechanisms by which heterologous immunity may be useful or detrimental when designing vaccines and to identify individuals potentially at risk for infection-related pathology. However, it is possible that a recent episode of acute IAV infection that has activated potentially cross-reactive IAV-M1-specific resident memory T cells in the tonsils may enhance the risk of developing EBV-induced AIM. It may be that something as simple as the annual conventional killed IAV vaccination, which generates neutralizing antibodies but does not normally activate IAV-specific CD8 T cells, might help prevent AIM upon EBV exposure by decreasing acute infection with IAV and thus decreasing the likelihood that IAV-specific resident memory cells accumulate in the tonsil.

## MATERIALS AND METHODS

### Subjects.

College students 18 to 30 years old who presented with symptoms of AIM participated in this study. Primary EBV infection was confirmed by a monospot test and the detection of EBV capsid-specific IgM in patient serum. Positive staining with HLA-A2.01^+^ tetramers loaded with influenza virus M1 peptide was used as an indication that these individuals had been exposed to IAV in the past (48). Patients provided up to eight blood samples (20 ml each) at entry (visit 1), 1 week (visit 2), 2 weeks (visit 3), 3 weeks (visit 4), 4 weeks (visit 5), 6 weeks (visit 6), 6 months (visit 7), and 1 year (visit 8). HLA typing was performed by screening with HLA-A2-specific MAb (BB7.2; EBioscience) staining and FACS analysis. Healthy, EBV-seropositive adult donors (HD-SP) >18 years old were used as controls. EBV infection was confirmed through the detection of EBV capsid antigen-specific (VCA) IgG antibodies in donor serum. Prior CMV infection was confirmed by CMV serology. Individual informed consent was obtained from each subject; the University of Massachusetts Medical School Human Subject Committee approved this study. The characteristics of our study subjects are summarized in Table S1.

### Grouping of AIM patients by severity of disease.

We developed a strategy to divide AIM patients into two groups, mild and severe AIM. Clinical measurements of disease severity collected in this study included patient-reported symptoms (malaise/fatigue, sore throat, headache, loss of appetite, myalgia, nausea, sweats, chills), physical findings on examination (adenopathy, pharyngitis, periorbital edema, hepatomegaly, and splenomegaly), with each symptom or sign rated on a scale of 1 to 10. Laboratory measurements of disease severity included the percentage of atypical lymphocytes and the CD4/CD8 ratio. A quantitative disease severity rating system that combined the severity of the most frequently observed physical finding (lymphadenopathy) with laboratory measurements of disease was calculated by using the sum of patient scores for peak percent adenopathy (clinical score by nurse: 0 to 10 × 10), the percentage of atypical lymphocytes on a peripheral smear, and the percent CD4/CD8 ratio in PBMCs determined with the formula {[2 − (CD4%/CD8%)]/2} × 100. Severe-AIM patients had total scores of ≥140, and mild-AIM patients had total scores of <140.

The combined clinical and laboratory disease severity score calculated in this manner is positively associated with the clinical disease severity score calculated by using all of the signs and symptoms in the cohort included in this study (*n* = 18, Spearman rho = 0.54, *P* = 0.02), as well as in the larger AIM cohort (*n* = 88, Spearman rho = 0.51, *P* < 0.001; data not shown).

### PBMC isolation and short-term T-cell culture.

Fresh (not frozen) PBMCs were isolated with Ficoll-Hypaque plus (Amersham Bioscience, Uppsala, Sweden) and stained with anti-CD8 antibody-coated microbeads (Miltenyi Biotech, Auburn, CA) in accordance with the manufacturer’s recommendations. Positive selection of CD8 lymphocytes was performed with the Miltenyi Biotech MACS system. Short-term (3-week) CD8 T-cell cultures were set up as previously described (48). Briefly, sorted CD8 lymphocytes were plated at a 5:1 ratio with 1 μM peptide-pulsed (for >1 h), irradiated T2 cells (CRL-1992; ATCC, Manassas, VA) that were washed free of excess peptide before being mixed with CD8 T cells. CD8 T-cell lines were fed with AIM V medium (Gibco) supplemented with 14% human AB serum (Nabi, Miami, FL, or Gemini, Woodland, CA), 16% MLA-144 supernatant (75), 10 U/ml recombinant IL-2 (BD), 1% l-glutamine (Gibco), 0.5% β-mercaptoethanol (Sigma, St. Louis, MO), 1% HEPES (HyClone, Logan, UT) every 3 to 4 days and restimulated with fresh peptide-pulsed T2 cells weekly for only 3 weeks. This culture method has been optimized so as not to skew the TCR repertoire from that present *in vivo* (43, 58, 60).

### HLA-A2.01-restricted peptides and MHC class I tetramers/pentamers.

The following peptides at >90% purity were purchased from 21st century (Waltham, MA): EBV-BMLF1_280–288_ (GLCTLVAML), EBV-BRLF1_109–117_ (YVLDHLIVV), EBV-EBNA-3A_509 to 604_ (SVRDRLARL), EBV-LMP-2_426–434_ (CLGGLLTMV), EBVLMP-2_329–337_ (LLWTLVVLL), CMV pp65_495–503_ (NLVPMVATV), IAV-M1_58–66_ (GILGFVFTL), IBV-NP_85−94_ (KLGEFYNQMM), and human endogenous tyrosinase_369–377_ (YMNGTMSQV). Tetramers were assembled with these peptide sequences for EBV-BMLF1, EBV-BRLF1, IAV-M1, CMV pp65, and tyrosinase by the tetramer facility at University of Massachusetts Medical School as previously described (48, 72). MHC class I pentamers for IAV-M1 and EBV-BMLF1 were purchased from ProImmune (Oxford, United Kingdom).

### Extracellular and intracellular staining.

Sorted CD8 T cells isolated from fresh PBMCs directly *ex vivo* or short-term-cultured CD8 T cells were plated at 10^6^/well in U-bottom 96-well plates (Sigma-Aldrich) and washed with staining buffer (phosphate-buffered saline, 2% fetal calf serum, 1% sodium azide). The first tetramer was incubated at room temperature for 20 min, and the excess was washed off before the addition of a second tetramer. In experiments where cells were costained with tetramers and/or pentamers, and with additional MAbs for surface markers (BD), such as CD3 (clone UCHT1), CD4 (clone RPA-T4), and CD8 (clone SK1), the tetramers were washed off prior to addition, and these MAbs were incubated for 20 min at room temperature as previously described (48). Cells stained in accordance with the manufacturer’s protocols were either fixed with FACS lysing solution or permeabilized with Cytofix/Cytoperm for intracellular assays. Anti-IFN-γ (clone B27) and anti-MIP-1β (clone D-21-1351) MAbs were used for ICS. All antibodies and reagents were purchased from BD. Flow cytometry was done with LSRII (Beckman Coulter, Inc., Fullerton, CA).

### EBV quantification.

Genomic DNA was extracted from enriched B cells with the DNeasy kit (Qiagen, Valencia, CA) (72, 76). Each sample was normalized to contain the DNA of a total of 10^6^ B cells. Samples were run in duplicate by using the recommended protocol accompanying a LightCycler EBV quantification kit (Roche Diagnostics, Indianapolis, IN). An internal positive control was supplied with this kit.

### TCR Vβ analysis.

Sorted CD8 T cells isolated from fresh PBMCs directly *ex vivo* were incubated for 20 min with IAV-M1-specific tetramer, which was then washed off. An additional 20-min incubation was performed with 24 TCR Vβ antibodies that cover >70% of the commonly used human Vβ types (IOTest Beta Mark TCR Vβ Repertoire kit; Beckman Coulter, Inc., Fullerton, CA). Samples were read on an LSRII (Beckman Coulter, Inc., Fullerton, CA). IMGT TCR gene nomenclature was used to define TCR Vβ types.

### TCR-tetramer blocking determination.

Sorted CD8 T cells isolated from fresh PBMCs directly *ex vivo* or short-term-cultured CD8 T cells from AIM patients were incubated with IAV-M1-, EBV-BM-, and EBV-BR-specific tetramers as described for extracellular staining. Since blocking of tetramer binding by the cross-reactive tetramer was a common event, we always used the following protocol for tetramer staining in order to accurately evaluate the frequency of cross-reactive CD8 T cells. Our panel for tetramer staining of CD8 T cells always included (i) each epitope-specific tetramer alone (IAV-M1, EBV-BM, EBV-BR, tyrosinase), (ii) generally non-cross-reactive control self-peptide specific tyrosinase tetramer with the epitope-specific tetramers together (IAV-M1, EBV-BM, EBV-BR) and (iii) a combination of double tetramer costaining with the following pairs: IAV-M1 plus EBV-BM, IAV-M1 plus EBV-BR and EBV-BM plus EBV-BR. Stained samples were read on an LSRII (Beckman Coulter, Inc., Fullerton, CA), and the percentage of tetramer^+^ cells and mean fluorescent intensity (MFI) of tetramer^+^ cells were analyzed with the FlowJo version 9.9.3 program. The estimated tetramer-blocking level for each tetramer was calculated by the formula (costained tetramer A MFI/alone tetramer A MFI) × (costained tetramer A%/alone tetramer A%) × 100 (Fig. 1; Fig. S2a and b).

### Statistical analyses.

Statistical analyses were performed with Prism version 6 (GraphPad Software, Inc.). The tests used included the Student *t* test, the linear regression test, and the nonparametric Mann-Whitney U test. The SDI was calculated as follows: *D* = Σ*n*(*n* − 1)/*N*(*N* − 1), where *D* is diversity, *n* is the number of individual clonotypes, and *N* is the number of unique clonotypes (58). The Student *t* test was used to compare two groups, and one-way ANOVA with Sidak’s or Tukey’s multiple-comparison test was used to compare more than two groups. To display pairwise correlations between variables of interest, we computed a correlation matrix by using the Pearson correlation coefficient. We then graphically displayed the matrix with the corrplot R package (https://cran.r-project.org/web/packages/corrplot/), which represents correlation values as circles with areas and color shades proportional to correlation values.

## References

[1] Taylor GS, Long HM, Brooks JM, Rickinson AB, Hislop AD 2015 The immunology of Epstein-Barr virus-induced disease. Annu Rev Immunol 33:787–821. doi:10.1146/annurev-immunol-032414-112326. 25706097

[2] Luzuriaga K, Sullivan JL 2010 Infectious mononucleosis. N Engl J Med 362:1993–2000. doi:10.1056/NEJMcp1001116. 20505178

[3] Balfour HH, Odumade OA, Schmeling DO, Mullan BD, Ed JA, Knight JA, Vezina HE, Thomas W, Hogquist KA (eds). 2013 Behavioral, virologic, and immunologic factors associated with acquisition and severity of primary Epstein-Barr virus infection in university students. J Infect Dis 207:80–88. doi:10.1093/infdis/jis646. 23100562 PMC3523797

[4] Cohen JI 2015 Primary immunodeficiencies associated with EBV disease. Curr Top Microbiol Immunol 390:241–265. doi:10.1007/978-3-319-22822-8_10. 26424649 PMC6349415

[5] Watkin LB, Mishra R, Gil A, Aslan N, Ghersi D, Luzuriaga K, Selin LK 2017 Unique influenza A cross-reactive memory CD8 TCR repertoire has a potential to protect against Epstein-Barr virus seroconversion. J Allergy Clin Immunol 140:1206–1210. doi:10.1016/j.jaci.2017.05.037. 28629751 PMC5669360

[6] Henle G, Henle W, Clifford P, Diehl V, Kafuko GW, Kirya BG, Klein G, Morrow RH, Munube GM, Pike P, Tukei PM, Ziegler JL 1969 Antibodies to Epstein-Barr virus in Burkitt’s lymphoma and control groups. J Natl Cancer Inst 43:1147–1157. 5353242

[7] Soulillou JP 2013 Missing links in multiple sclerosis etiology. A working connecting hypothesis. Med Hypotheses 80:509–516. doi:10.1016/j.mehy.2013.01.036. 23466062

[8] Moss DJ, Burrows SR, Silins SL, Misko I, Khanna R 2001 The immunology of Epstein-Barr virus infection. Philos Trans R Soc Lond B Biol Sci 356:475–488. doi:10.1098/rstb.2000.0784. 11313006 PMC1088439

[9] Callan MF, Tan L, Annels N, Ogg GS, Wilson JD, O’Callaghan CA, Steven N, McMichael AJ, Rickinson AB 1998 Direct visualization of antigen-specific CD8^+^ T cells during the primary immune response to Epstein-Barr virus in vivo. J Exp Med 187:1395–1402. doi:10.1084/jem.187.9.1395. 9565632 PMC2212279

[10] Catalina MD, Sullivan JL, Bak KR, Luzuriaga K 2001 Differential evolution and stability of epitope-specific CD8(+) T-cell responses in EBV infection. J Immunol 167:4450–4457. doi:10.4049/jimmunol.167.8.4450. 11591771

[11] Dreyfus DH 2017 Gene sharing between Epstein-Barr virus and human immune response genes. Immunol Res 65:37–45. doi:10.1007/s12026-016-8814-x. 27421718

[12] Ressing ME, van Gent M, Gram AM, Hooykaas MJG, Piersma SJ, Wiertz EJHJ 2015 Immune evasion by Epstein-Barr virus. Curr Top Microbiol Immunol 391:355–381. doi:10.1007/978-3-319-22834-1_12. 26428381

[13] Dunmire SK, Odumade OA, Porter JL, Reyes-Genere J, Schmeling DO, Bilgic H, Fan D, Baechler EC, Balfour HH, Hogquist KA 2014 Primary EBV infection induces an expression profile distinct from other viruses but similar to hemophagocytic syndromes. PLoS One 9:e85422. doi:10.1371/journal.pone.0085422. 24465555 PMC3894977

[14] Panikkar A, Smith C, Hislop A, Tellam N, Dasari V, Hogquist KA, Wykes M, Moss DJ, Rickinson A, Balfour HH, Khanna R 2015 Cytokine-mediated loss of blood dendritic cells during Epstein-Barr virus-associated acute infectious mononucleosis: implication for immune dysregulation. J Infect Dis 212:1957–1961. doi:10.1093/infdis/jiv340. 26080368

[15] Rehermann B, Shin EC 2005 Private aspects of heterologous immunity. J Exp Med 201:667–670. doi:10.1084/jem.20050220. 15753200 PMC2212842

[16] Burrows SR, Silins SL, Khanna R, Burrows JM, Rischmueller M, McCluskey J, Moss DJ 1997 Cross-reactive memory T cells for Epstein-Barr virus augment the alloresponse to common human leukocyte antigens: degenerate recognition of major histocompatibility complex-bound peptide by T cells and its role in alloreactivity. Eur J Immunol 27:1726–1736. doi:10.1002/eji.1830270720. 9247584

[17] Selin LK, Varga SM, Wong IC, Welsh RM 1998 Protective heterologous antiviral immunity and enhanced immunopathogenesis mediated by memory T-cell populations. J Exp Med 188:1705–1715. doi:10.1084/jem.188.9.1705. 9802982 PMC2212518

[18] Furman D, Jojic V, Sharma S, Shen-Orr SS, Angel CJL, Onengut-Gumuscu S, Kidd BA, Maecker HT, Concannon P, Dekker CL, Thomas PG, Davis MM 2015 Cytomegalovirus infection enhances the immune response to influenza. Sci Transl Med 7:281ra43. doi:10.1126/scitranslmed.aaa2293. PMC4505610 25834109

[19] Sharma S, Thomas PG 2014 The two faces of heterologous immunity: protection or immunopathology. J Leukoc Biol 95:405–416. doi:10.1189/jlb.0713386. 24212098 PMC3923083

[20] Cornberg M, Wedemeyer H 2016 Hepatitis C virus infection from the perspective of heterologous immunity. Curr Opin Virol 16:41–48. doi:10.1016/j.coviro.2016.01.005. 26826311

[21] Welsh RM, Che JW, Brehm MA, Selin LK 2010 Heterologous immunity between viruses. Immunol Rev 235:244–266. doi:10.1111/j.0105-2896.2010.00897.x. 20536568 PMC2917921

[22] Welsh RM, Selin LK 2002 No one is naive: the significance of heterologous T-cell immunity. Nat Rev Immunol 2:417–426. doi:10.1038/nri820. 12093008

[23] Selin LK, Nahill SR, Welsh RM 1994 Cross-reactivities in memory cytotoxic T lymphocyte recognition of heterologous viruses. J Exp Med 179:1933–1943. doi:10.1084/jem.179.6.1933. 8195718 PMC2191532

[24] Brehm MA, Pinto AK, Daniels KA, Schneck JP, Welsh RM, Selin LK 2002 T-cell immunodominance and maintenance of memory regulated by unexpectedly cross-reactive pathogens. Nat Immunol 3:627–634. doi:10.1038/ni806. 12055626

[25] Zhang S, Bakshi RK, Suneetha PV, Fytili P, Antunes DA, Vieira GF, Jacobs R, Klade CS, Manns MP, Kraft ARM, Wedemeyer H, Schlaphoff V, Cornberg M 2015 Frequency, private specificity, and cross-reactivity of preexisting hepatitis C virus (HCV)-specific CD8^+^ T cells in HCV-seronegative individuals: implications for vaccine responses. J Virol 89:8304–8317. doi:10.1128/JVI.00539-15. 26041301 PMC4524240

[26] Chen HD, Fraire AE, Joris I, Welsh RM, Selin LK 2003 Specific history of heterologous virus infections determines anti-viral immunity and immunopathology in the lung. Am J Pathol 163:1341–1355. doi:10.1016/S0002-9440(10)63493-1. 14507643 PMC1868309

[27] Selin LK, Vergilis K, Welsh RM, Nahill SR 1996 Reduction of otherwise remarkably stable virus-specific cytotoxic T lymphocyte memory by heterologous viral infections. J Exp Med 183:2489–2499. doi:10.1084/jem.183.6.2489. 8676069 PMC2192604

[28] Selin LK, Lin MY, Kraemer KA, Pardoll DM, Schneck JP, Varga SM, Santolucito PA, Pinto AK, Welsh RM 1999 Attrition of T-cell memory: selective loss of LCMV epitope-specific memory CD8 T cells following infections with heterologous viruses. Immunity 11:733–742. doi:10.1016/S1074-7613(00)80147-8. 10626895

[29] Cornberg M, Chen AT, Wilkinson LA, Brehm MA, Kim SK, Calcagno C, Ghersi D, Puzone R, Celada F, Welsh RM, Selin LK 2006 Narrowed TCR repertoire and viral escape as a consequence of heterologous immunity. J Clin Invest 116:1443–1456. doi:10.1172/JCI27804. 16614754 PMC1435724

[30] Nie S, Cornberg M, Selin LK 2009 Resistance to vaccinia virus is less dependent on TNF under conditions of heterologous immunity. J Immunol 183:6554–6560. doi:10.4049/jimmunol.0902156. 19846867 PMC3242700

[31] Chen HD, Fraire AE, Joris I, Brehm MA, Welsh RM, Selin LK 2001 Memory CD8^+^ T cells in heterologous antiviral immunity and immunopathology in the lung. Nat Immunol 2:1067–1076. doi:10.1038/ni727. 11668342

[32] Reese TA, Bi K, Kambal A, Filali-Mouhim A, Beura LK, Bürger MC, Pulendran B, Sekaly RP, Jameson SC, Masopust D, Haining WN, Virgin HW 2016 Sequential infection with common pathogens promotes human-like immune gene expression and altered vaccine response. Cell Host Microbe 19:713–719. doi:10.1016/j.chom.2016.04.003. 27107939 PMC4896745

[33] Wlodarczyk MF, Kraft AR, Chen HD, Kenney LL, Selin LK 2013 Anti-IFN-γ and peptide-tolerization therapies inhibit acute lung injury induced by cross-reactive influenza A-specific memory T cells. J Immunol 190:2736–2746. doi:10.4049/jimmunol.1201936. 23408839 PMC3594402

[34] Urbani S, Amadei B, Fisicaro P, Pilli M, Missale G, Bertoletti A, Ferrari C 2005 Heterologous T-cell immunity in severe hepatitis C virus infection. J Exp Med 201:675–680. doi:10.1084/jem.20041058. 15753202 PMC2212827

[35] Welsh RM, Rothman AL 2003 Dengue immune response: low affinity, high febrility. Nat Med 9:820–822. doi:10.1038/nm0703-820. 12835692

[36] Wen J, Tang WW, Sheets N, Ellison J, Sette A, Kim K, Shresta S 2017 Identification of Zika virus epitopes reveals immunodominant and protective roles for dengue virus cross-reactive CD8^+^ T cells. Nat Microbiol 2:17036. doi:10.1038/nmicrobiol.2017.36. 28288094 PMC5918137

[37] Haanen JB, Wolkers MC, Kruisbeek AM, Schumacher TN 1999 Selective expansion of cross-reactive CD8(+) memory T cells by viral variants. J Exp Med 190:1319–1328. doi:10.1084/jem.190.9.1319. 10544203 PMC2195685

[38] Effros RB, Doherty PC, Gerhard W, Bennink J 1977 Generation of both cross-reactive and virus-specific T-cell populations after immunization with serologically distinct influenza A viruses. J Exp Med 145:557–568. doi:10.1084/jem.145.3.557. 233901 PMC2180700

[39] Epstein SL 2006 Prior H1N1 influenza infection and susceptibility of Cleveland Family Study participants during the H2N2 pandemic of 1957: an experiment of nature. J Infect Dis 193:49–53. doi:10.1086/498980. 16323131

[40] Benn CS, Netea MG, Selin LK, Aaby P 2013 A small jab—a big effect: nonspecific immunomodulation by vaccines. Trends Immunol 34:431–439. doi:10.1016/j.it.2013.04.004. 23680130

[41] Steenhuis TJ, van Aalderen WMC, Bloksma N, Nijkamp FP, van der Laag J, van Loveren H, Rijkers GT, Kuis W, Hoekstra MO 2008 Bacille-Calmette-Guerin vaccination and the development of allergic disease in children: a randomized, prospective, single-blind study. Clin Exp Allergy 38:79–85. doi:10.1111/j.1365-2222.2007.02859.x. 17956585

[42] Rickinson AB, Kieff E 1996 Epstein-Barr virus, p 2397–2446. *In* Fields BN, Knipe DM, Howley PM (ed), Fields virology, vol 2 Lippincott-Raven, Philadelphia, PA.

[43] Song I-Y, Gil A, Mishra R, Ghersi D, Selin LK, Stern LJ 2017 Broad TCR repertoire and diverse structural solutions for recognition of an immunodominant CD8^+^ T-cell epitope. Nat Struct Mol Biol 24:395–406. doi:10.1038/nsmb.3383. 28250417 PMC5383516

[44] Dash P, Fiore-Gartland AJ, Hertz T, Wang G, Sharma S, Souquette A, Crawford JC, Clemens EB, Nguyen THO, Kedzierska K, La Gruta NL, Bradley P, Thomas PG 2017 Quantifiable predictive features define epitope-specific T-cell receptor repertoire. Nature 547:89–93. doi:10.1038/nature22383. 28636592 PMC5616171

[45] Glanville J, Huang H, Nau A, Hatton O, Wagar LE, Rubelt F, Ji X, Han A, Krams SM, Pettus C, Haas N, Arlehamn CSL, Sette A, Boyd SD, Scriba TJ, Martinez OM, Davis MM 2017 Identifying specificity groups in the T-cell receptor repertoire. Nature 547:94–98. doi:10.1038/nature22976. 28636589 PMC5794212

[46] Chen G, Yang X, Ko A, Sun X, Gao M, Zhang Y, Shi A, Mariuzza RA, Weng NP 2017 Sequence and structural analyses reveal distinct and highly diverse human CD8^+^ TCR repertoires to immunodominant viral antigens. Cell Rep 19:569–583. doi:10.1016/j.celrep.2017.03.072. 28423320 PMC5472051

[47] Attaf M, Sewell AK 2016 Disease etiology and diagnosis by TCR repertoire analysis goes viral. Eur J Immunol 46:2516–2519. doi:10.1002/eji.201646649. 27813075

[48] Clute SC, Watkin LB, Cornberg M, Naumov YN, Sullivan JL, Luzuriaga K, Welsh RM, Selin LK 2005 Cross-reactive influenza virus-specific CD8^+^ T cells contribute to lymphoproliferation in Epstein-Barr virus-associated infectious mononucleosis. J Clin Invest 115:3602–3612. doi:10.1172/JCI25078. 16308574 PMC1288832

[49] Cornberg M, Clute SC, Watkin LB, Saccoccio FM, Kim SK, Naumov YN, Brehm MA, Aslan N, Welsh RM, Selin LK 2010 CD8 T-cell cross-reactivity networks mediate heterologous immunity in human EBV and murine vaccinia virus infections. J Immunol 184:2825–2838. doi:10.4049/jimmunol.0902168. 20164414 PMC3253758

[50] Selin LK, Wlodarczyk MF, Kraft AR, Nie S, Kenney LL, Puzone R, Celada F 2011 Heterologous immunity: immunopathology, autoimmunity and protection during viral infections. Autoimmunity 44:328–347. doi:10.3109/08916934.2011.523277. 21250837 PMC3633594

[51] Nie S, Lin SJ, Kim SK, Welsh RM, Selin LK 2010 Pathological features of heterologous immunity are regulated by the private specificities of the immune repertoire. Am J Pathol 176:2107–2112. doi:10.2353/ajpath.2010.090656. 20348239 PMC2861077

[52] Chen AT, Cornberg M, Gras S, Guillonneau C, Rossjohn J, Trees A, Emonet S, de la Torre JC, Welsh RM, Selin LK 2012 Loss of anti-viral immunity by infection with a virus encoding a cross-reactive pathogenic epitope. PLoS Pathog 8:e1002633. doi:10.1371/journal.ppat.1002633. 22536152 PMC3334890

[53] Kim SK, Cornberg M, Wang XZ, Chen HD, Selin LK, Welsh RM 2005 Private specificities of CD8 T-cell responses control patterns of heterologous immunity. J Exp Med 201:523–533. doi:10.1084/jem.20041337. 15710651 PMC2213046

[54] Kenney LL, Cornberg M, Chen AT, Emonet S, de la Torre JC, Selin LK 2015 Increased immune response variability during simultaneous viral coinfection leads to unpredictability in CD8 T-cell immunity and pathogenesis. J Virol 89:10786–10801. doi:10.1128/JVI.01432-15. 26269191 PMC4621125

[55] Dolton G, Lissina A, Skowera A, Ladell K, Tungatt K, Jones E, Kronenberg-Versteeg D, Akpovwa H, Pentier JM, Holland CJ, Godkin AJ, Cole DK, Neller MA, Miles JJ, Price DA, Peakman M, Sewell AK 2014 Comparison of peptide-major histocompatibility complex tetramers and dextramers for the identification of antigen-specific T cells. Clin Exp Immunol 177:47–63. doi:10.1111/cei.12339. 24673376 PMC4089154

[56] Choo JAL, Liu J, Toh X, Grotenbreg GM, Ren EC 2014 The immunodominant influenza A virus M1_58–66_ cytotoxic T lymphocyte epitope exhibits degenerate class I major histocompatibility complex restriction in humans. J Virol 88:10613–10623. doi:10.1128/JVI.00855-14. 24990997 PMC4178881

[57] Laugel B, van den Berg HA, Gostick E, Cole DK, Wooldridge L, Boulter J, Milicic A, Price DA, Sewell AK 2007 Different T-cell receptor affinity thresholds and CD8 coreceptor dependence govern cytotoxic T lymphocyte activation and tetramer binding properties. J Biol Chem 282:23799–23810. doi:10.1074/jbc.M700976200. 17540778

[58] Naumov YN, Naumova EN, Clute SC, Watkin LB, Kota K, Gorski J, Selin LK 2006 Complex T-cell memory repertoires participate in recall responses at extremes of antigenic load. J Immunol 177:2006–2014. doi:10.4049/jimmunol.177.3.2006. 16849515

[59] Venturi V, Kedzierska K, Turner SJ, Doherty PC, Davenport MP 2007 Methods for comparing the diversity of samples of the T-cell receptor repertoire. J Immunol Methods 321:182–195. doi:10.1016/j.jim.2007.01.019. 17337271

[60] Clute SC, Naumov YN, Watkin LB, Aslan N, Sullivan JL, Thorley-Lawson DA, Luzuriaga K, Welsh RM, Puzone R, Celada F, Selin LK 2010 Broad cross-reactive TCR repertoires recognizing dissimilar Epstein-Barr and influenza A virus epitopes. J Immunol 185:6753–6764. doi:10.4049/jimmunol.1000812. 21048112 PMC3738202

[61] Hombrink P, Raz Y, Kester MG, de Boer R, Weißbrich B, von dem Borne PA, Busch DH, Schumacher TN, Falkenburg JH, Heemskerk MH 2013 Mixed functional characteristics correlating with TCR-ligand k_off_-rate of MHC-tetramer reactive T cells within the naive T-cell repertoire. Eur J Immunol 43:3038–3050. doi:10.1002/eji.201343397. 23893393

[62] Han A, Glanville J, Hansmann L, Davis MM 2014 Linking T-cell receptor sequence to functional phenotype at the single-cell level. Nat Biotechnol 32:684–692. doi:10.1038/nbt.2938. 24952902 PMC4337815

[63] Young HA, Hardy KJ 1995 Role of interferon-gamma in immune cell regulation. J Leukoc Biol 58:373–381. 7561512

[64] Gil A, Yassai MB, Naumov YN, Selin LK 2015 Narrowing of human influenza A virus-specific T-cell receptor α and β repertoires with increasing age. J Virol 89:4102–4116. doi:10.1128/JVI.03020-14. 25609818 PMC4442365

[65] Jing L, Laing KJ, Dong L, Russell RM, Barlow RS, Haas JG, Ramchandani MS, Johnston C, Buus S, Redwood AJ, White KD, Mallal SA, Phillips EJ, Posavad CM, Wald A, Koelle DM 2016 Extensive CD4 and CD8 T-cell cross-reactivity between alphaherpesviruses. J Immunol 196:2205–2218. doi:10.4049/jimmunol.1502366. 26810224 PMC4761520

[66] Lossius A, Johansen JN, Vartdal F, Robins H, Jūratė Šaltytė B, Holmøy T, Olweus J 2014 High-throughput sequencing of TCR repertoires in multiple sclerosis reveals intrathecal enrichment of EBV-reactive CD8^+^ T cells. Eur J Immunol 44:3439–3452. doi:10.1002/eji.201444662. 25103993

[67] Sloan-Lancaster J, Allen PM 1996 Altered peptide ligand-induced partial T-cell activation: molecular mechanisms and role in T-cell biology. Annu Rev Immunol 14:1–27. doi:10.1146/annurev.immunol.14.1.1. 8717505

[68] Guy CS, Vignali DAA 2009 Organization of proximal signal initiation at the TCR:CD3 complex. Immunol Rev 232:7–21. doi:10.1111/j.1600-065X.2009.00843.x. 19909352 PMC2845712

[69] Guy CS, Vignali KM, Temirov J, Bettini ML, Overacre AE, Smeltzer M, Zhang H, Huppa JB, Tsai YH, Lobry C, Xie J, Dempsey PJ, Crawford HC, Aifantis I, Davis MM, Vignali DAA 2013 Distinct TCR signaling pathways drive proliferation and cytokine production in T cells. Nat Immunol 14:262–270. doi:10.1038/ni.2538. 23377202 PMC3577985

[70] van den Berg HA, Wooldridge L, Laugel B, Sewell AK 2007 Coreceptor CD8-driven modulation of T-cell antigen receptor specificity. J Theor Biol 249:395–408. doi:10.1016/j.jtbi.2007.08.002. 17869274 PMC6485485

[71] Laugel B, Cole DK, Clement M, Wooldridge L, Price DA, Sewell AK 2011 The multiple roles of the CD8 coreceptor in T-cell biology: opportunities for the selective modulation of self-reactive cytotoxic T cells. J Leukoc Biol 90:1089–1099. doi:10.1189/jlb.0611316. 21954283

[72] Greenough TC, Campellone SC, Brody R, Jain S, Sanchez-Merino V, Somasundaran M, Luzuriaga K 2010 Programmed Death-1 expression on Epstein-Barr virus specific CD8^+^ T cells varies by stage of infection, epitope specificity, and T-cell receptor usage. PLoS One 5:e12926. doi:10.1371/journal.pone.0012926. 20886079 PMC2944873

[73] Cornberg M, Kenney LL, Chen AT, Waggoner SN, Kim SK, Dienes HP, Welsh RM, Selin LK 2013 Clonal exhaustion as a mechanism to protect against severe immunopathology and death from an overwhelming CD8 T-cell response. Front Immunol 4:475. doi:10.3389/fimmu.2013.00475. 24391647 PMC3869045

[74] Dunmire SK, Grimm JM, Schmeling DO, Balfour HH, Hogquist KA 2015 The incubation period of primary Epstein-Barr virus infection: viral dynamics and immunologic events. PLoS Pathog 11:e1005286. doi:10.1371/journal.ppat.1005286. 26624012 PMC4666617

[75] Rabin H, Hopkins RF, Ruscetti FW, Neubauer RH, Brown RL, Kawakami TG 1981 Spontaneous release of a factor with properties of T-cell growth factor from a continuous line of primate tumor T cells. J Immunol 127:1852–1856. 6975300

[76] Weiss ER, Alter G, Ogembo JG, Henderson JL, Tabak B, Bakiş Y, Somasundaran M, Garber M, Selin L, Luzuriaga K 2017 High Epstein-Barr virus load and genomic diversity are associated with generation of gp350-specific neutralizing antibodies following acute infectious mononucleosis. J Virol 91:e01562-16. doi:10.1128/JVI.01562-16. 27733645 PMC5165192

