# Supplementary material for: Severity of Acute Infectious Mononucleosis Correlates with Cross-Reactive Influenza CD8 T-Cell Receptor Repertoires
Source: mBio. 2017 Dec 5;8(6):e01841-17. doi: 10.1128/mBio.01841-17 (PMC5717389; doi:10.1128/mBio.01841-17)
Supplement: TABLE S1 [file mbo006173603st1.docx]

**Supplemental Table 1: Characteristics of the study populations**

| **Study Population** | **HD-SP** | **AIM patients**  **(all)** | **Severe-AIM**  **patients ≠** | **Mild-AIM**  **patients ≠** |
| --- | --- | --- | --- | --- |
| **Median age years (range)** | **20**  **(18-50)** | **20**  **(18-30)** | **20**  **(19-21)** | **20**  **(18-30)** |
| **Number of subjects (n)** | **17** | **32** | **16** | **16** |
| **Sex (F/M)** | **10/7** | **17/15** | **8/8** | **9/7** |
| **Mean adenopathy score**  **± SEM** | **NA** | **4.9±0.7** | **6.5±0.6^*^** | **3.3±0.8^*^** |
| **Mean % atypical lymphocytes ± SEM** | **NA** | **34.3±3.7** | **44±4.2**** | **23±3.8**** |
| **Mean CD4/CD8 ratio ± SEM** | **3.3±0.6** | **0.9±0.1** | **0.6±0.08***** | **1.4±0.1***** |

**≠: When severe-AIM group is compared to mild-AIM : *p: 0.01, **p: 0.002 ,**

*****p: 0.001 (Student’s t test)**
