# Supplementary figures and images for: Severity of Acute Infectious Mononucleosis Correlates with Cross-Reactive Influenza CD8 T-Cell Receptor Repertoires
Source: mBio. 2017 Dec 5;8(6):e01841-17. doi: 10.1128/mBio.01841-17 (PMC5717389; doi:10.1128/mBio.01841-17)

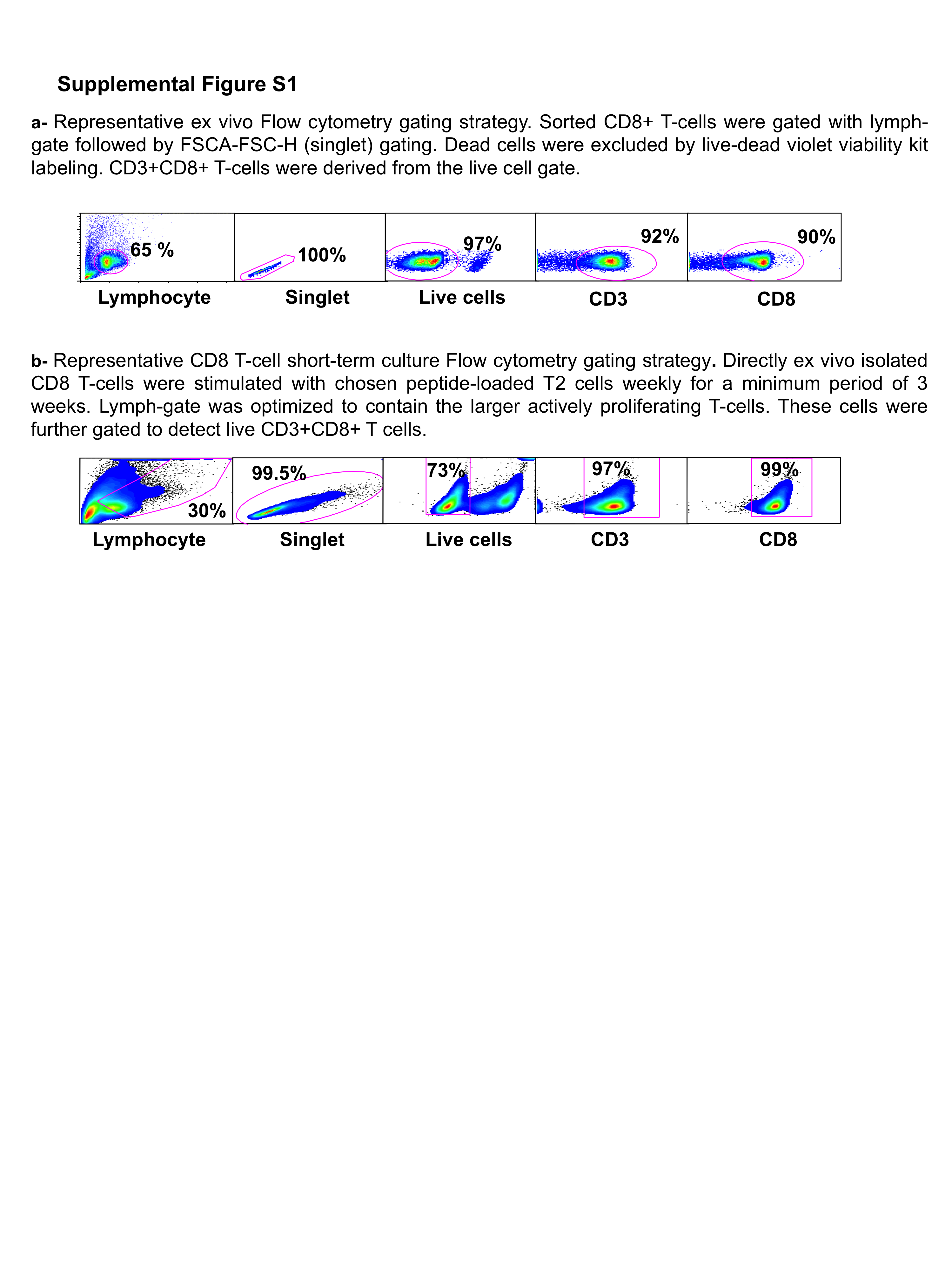

Supplement: FIG S1 [file mbo006173603sf1.tif]

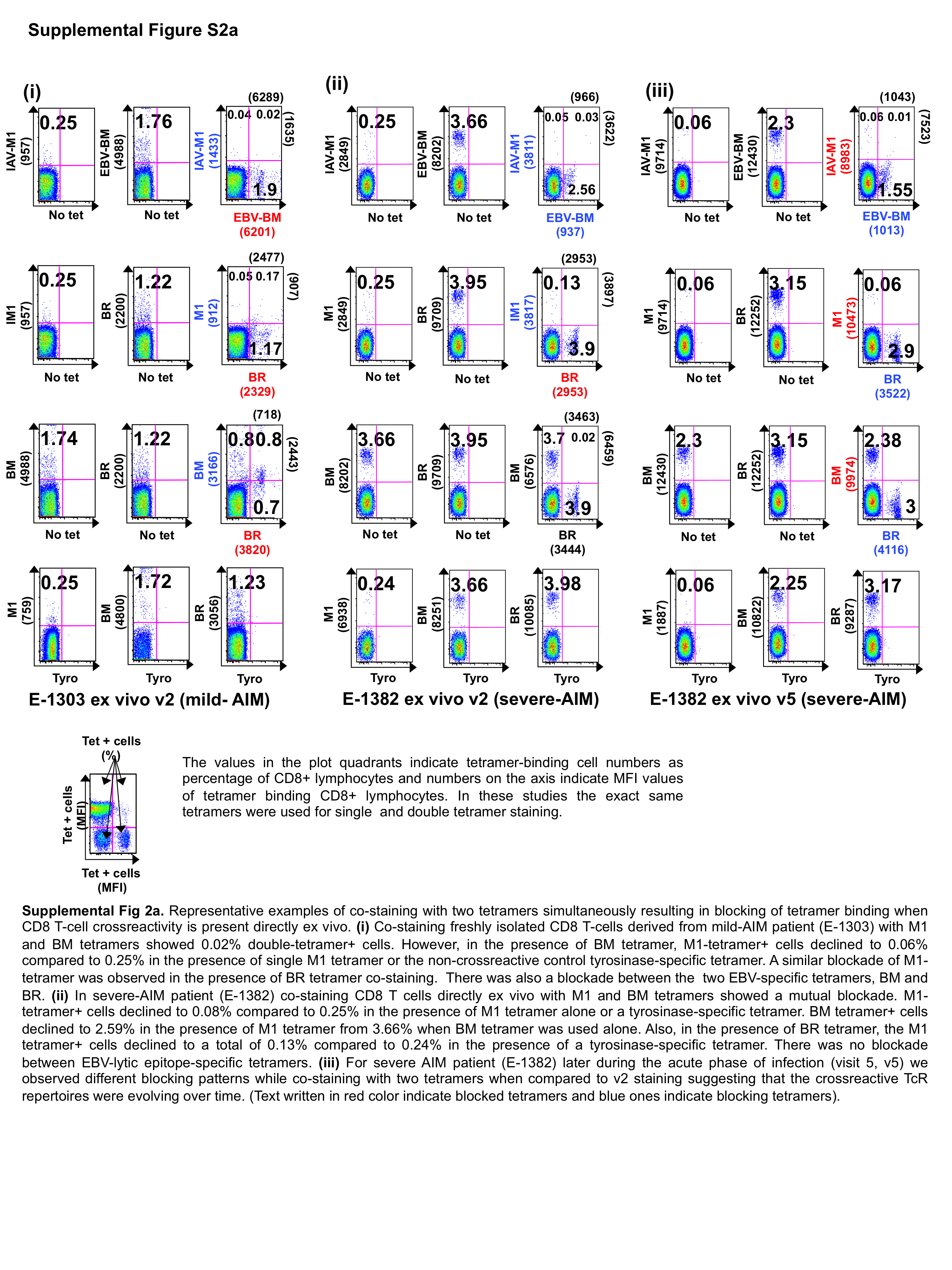

Supplement: FIG S2a [file mbo006173603sf2.tif]

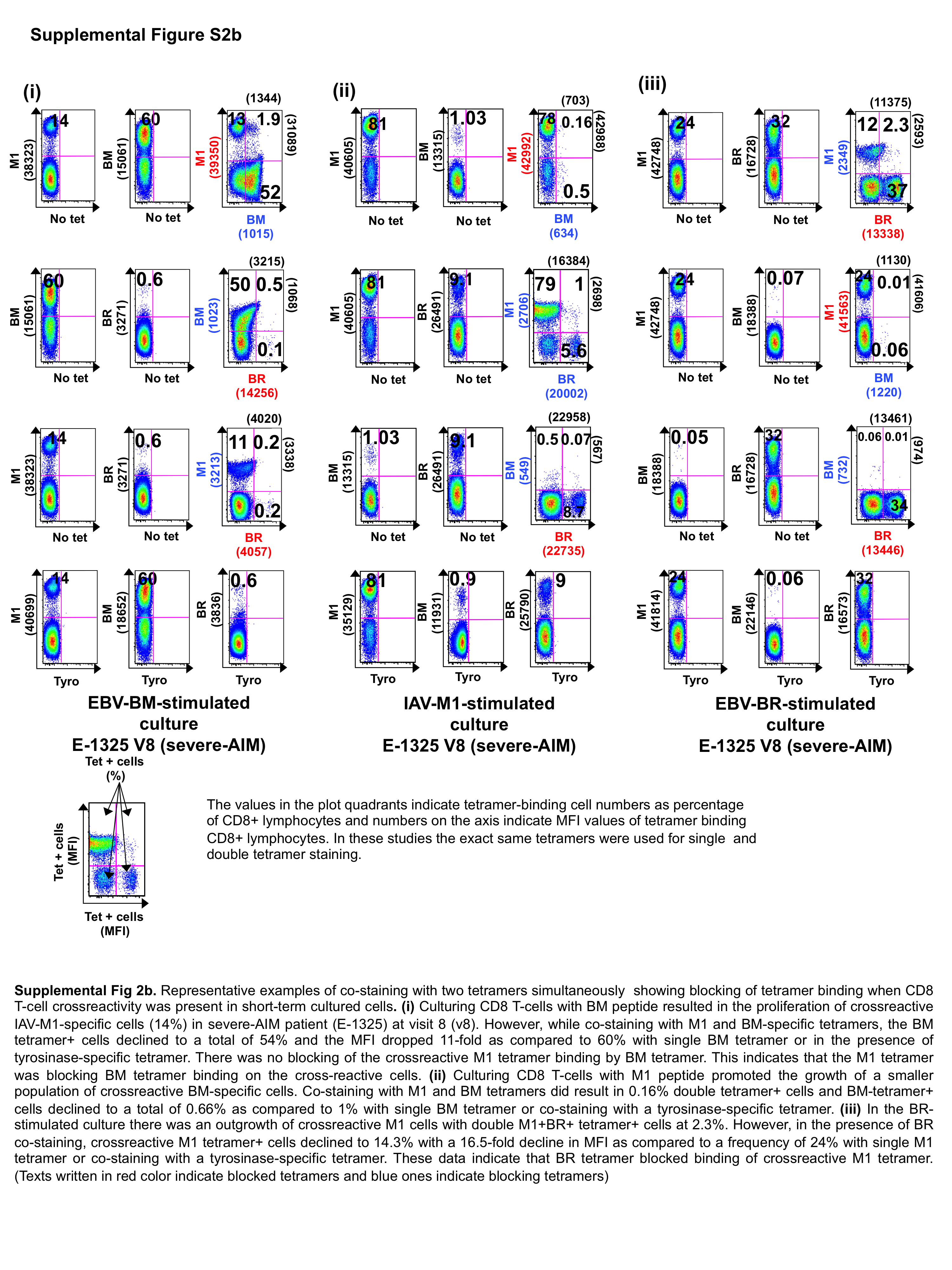

Supplement: FIG S2b [file mbo006173603sf3.tif]

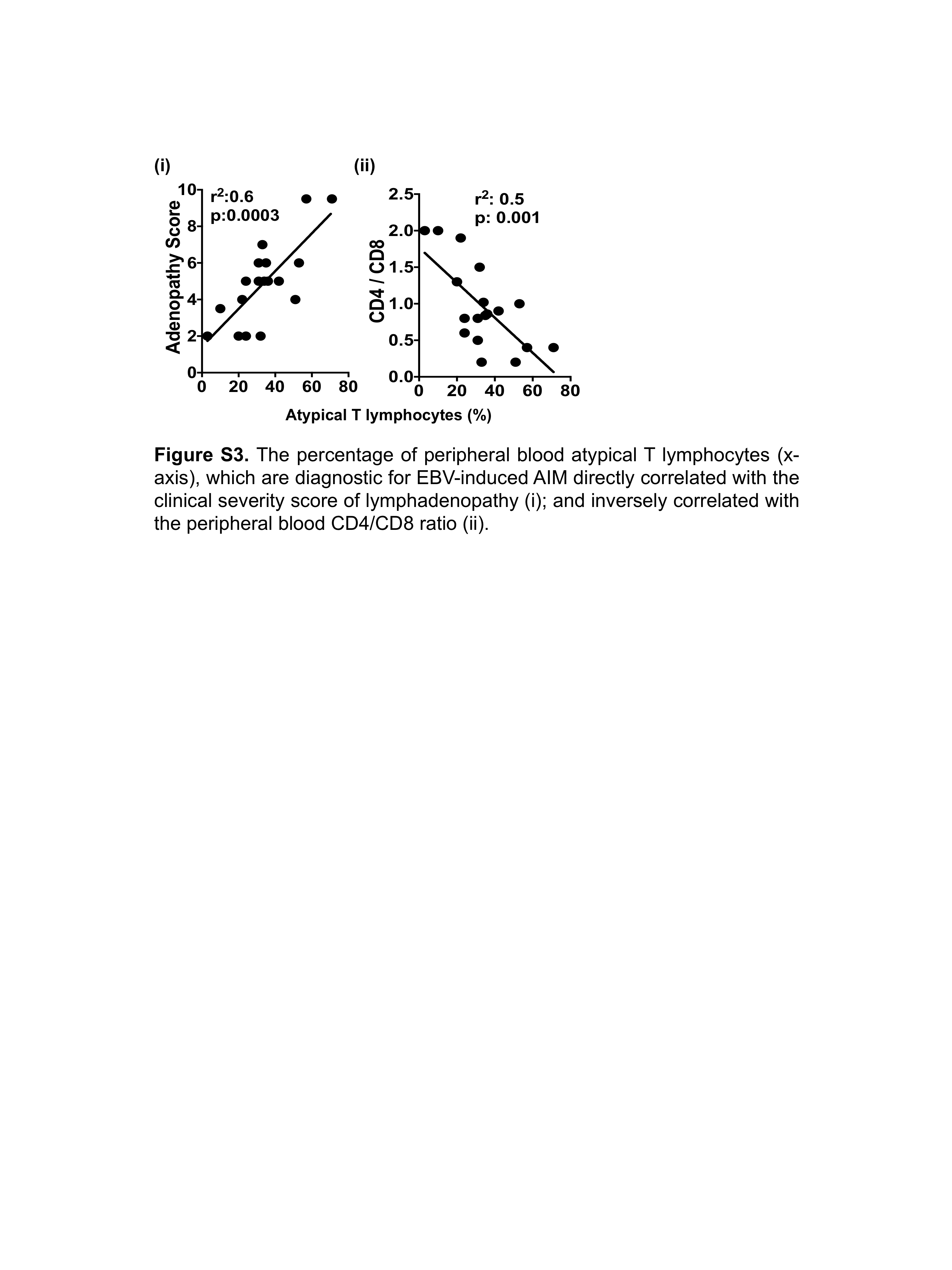

Supplement: FIG S3 [file mbo006173603sf4.tif]

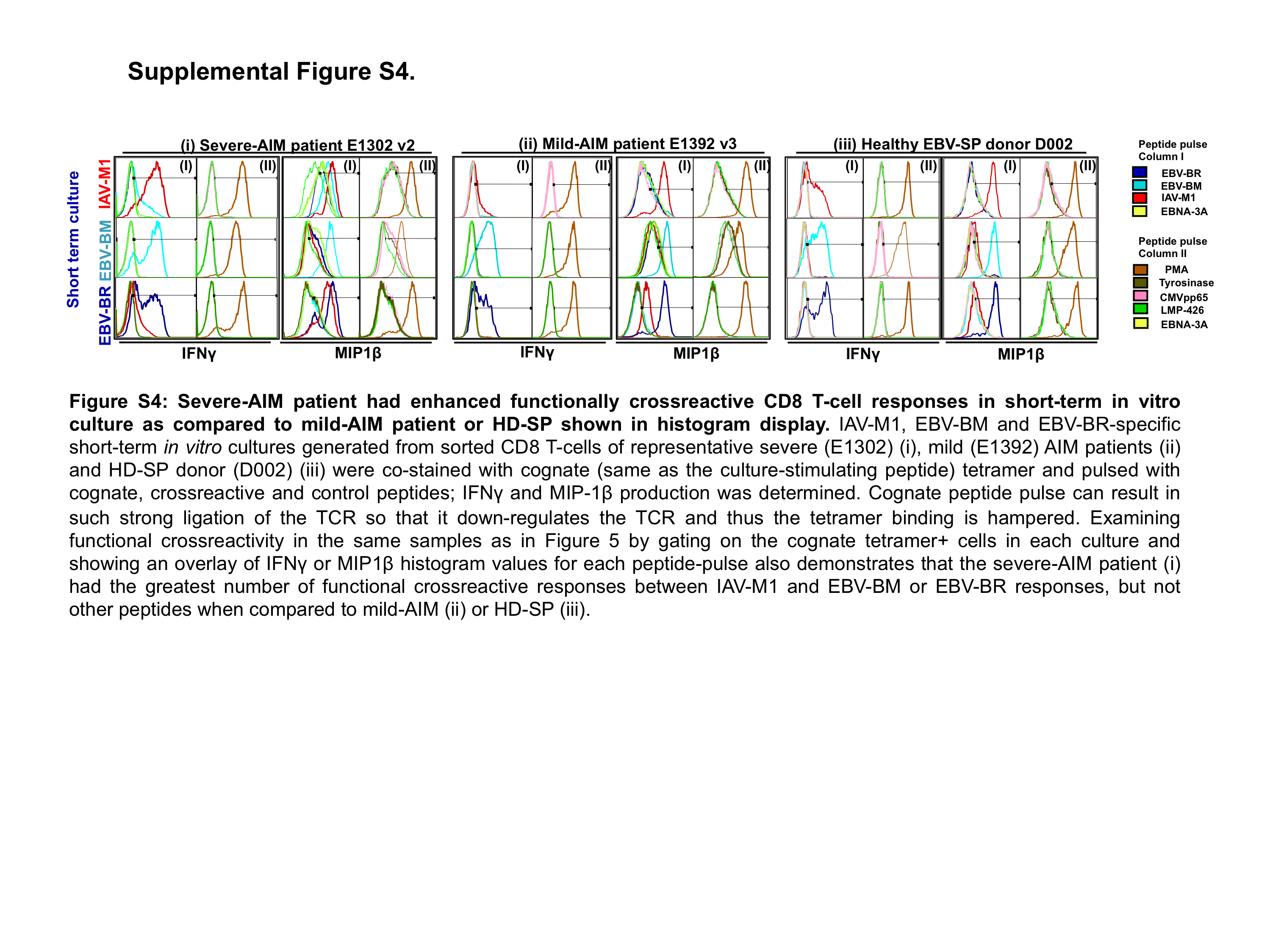

Supplement: FIG S4 [file mbo006173603sf5.tif]

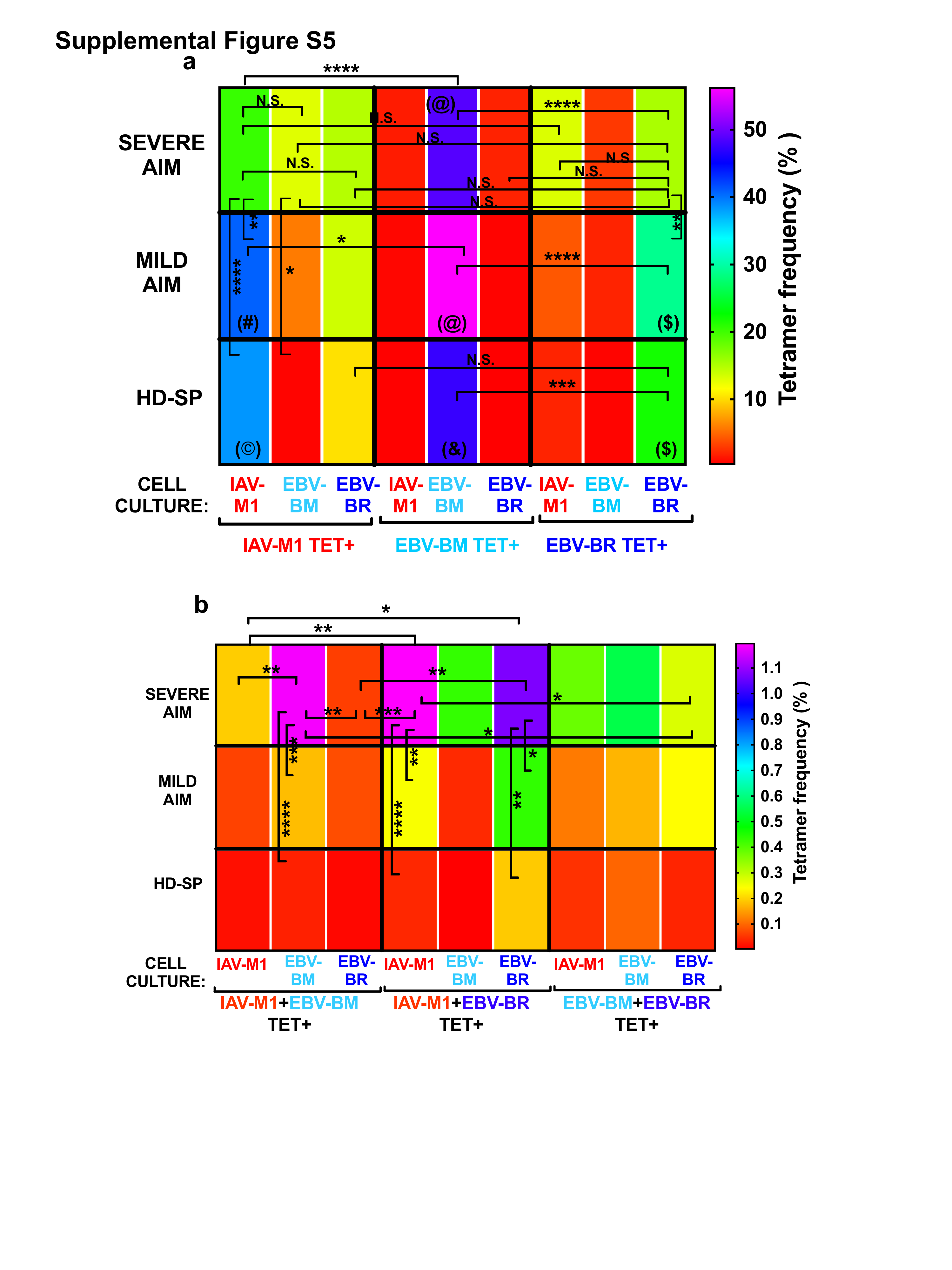

Supplement: FIG S5 [file mbo006173603sf6.tif]

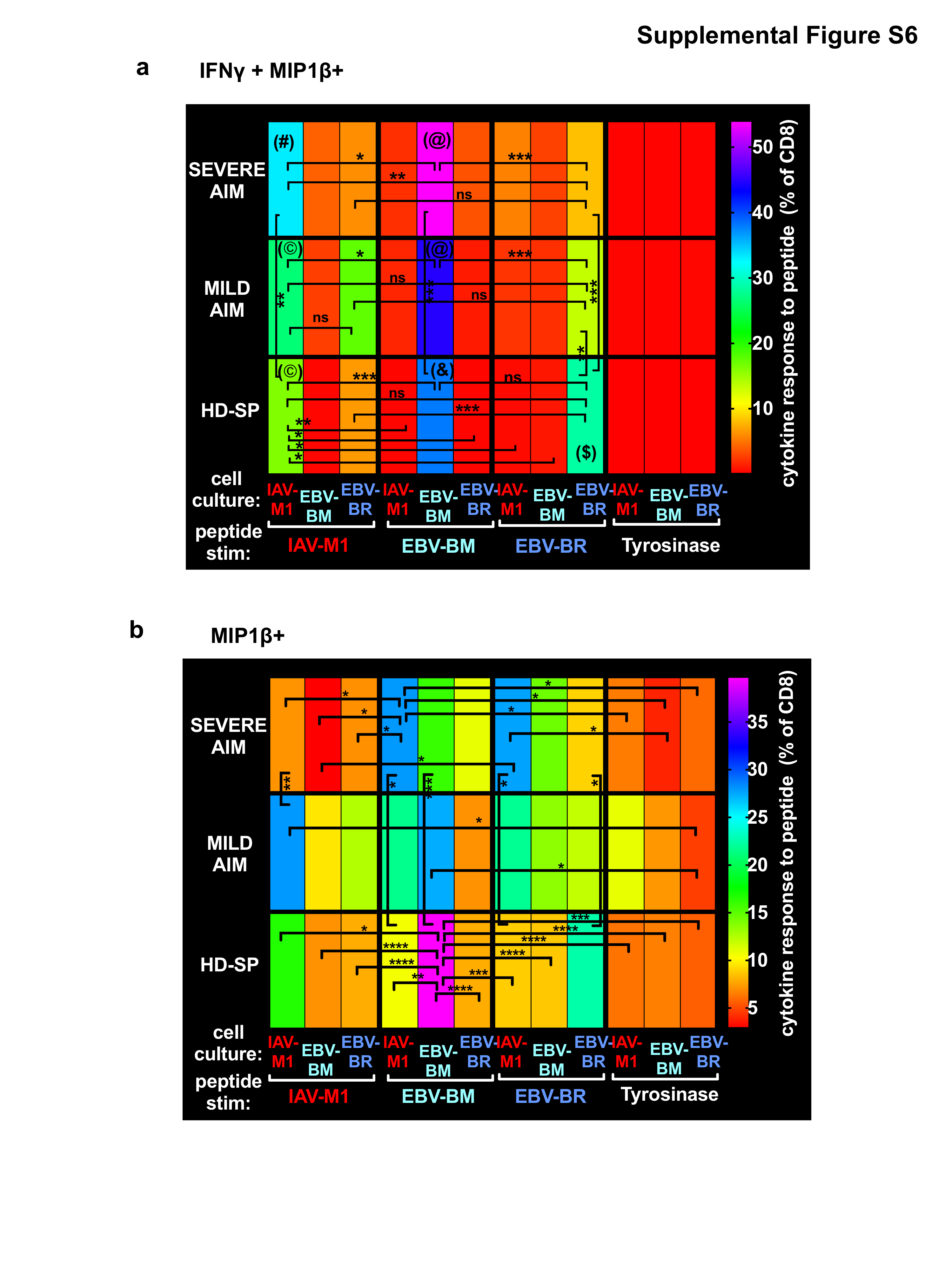

Supplement: FIG S6 [file mbo006173603sf7.tif]
