# Supplementary material for: Severity of Acute Infectious Mononucleosis Correlates with Cross-Reactive Influenza CD8 T-Cell Receptor Repertoires
Source: mBio. 2017 Dec 5;8(6):e01841-17. doi: 10.1128/mBio.01841-17 (PMC5717389; doi:10.1128/mBio.01841-17)
Supplement: TABLE S2 [file mbo006173603st2.docx]

Supplemental Table 2. Factors associated with increased relative risk of Severe-AIM diagnosis^#^

| Data variables | Relative risk | P value |
| --- | --- | --- |
| IAV-M1 tetramer+ ≥ 0.36 | 4.9 | 0.049* |
| IAV-M1+EBV-BM tetramer+ ≥ 0.1 | 5.8 | 0.02* |
| EBV-BM tetramer+ ≥ 2.2 | 3.0 | 0.15 |
| EBV-BR tetramer+ ≥ 2.0 | 1.1 | >0.9999 |
| IAV-M1+EBV-BR tetramer+ ≥ 0.1 | 1.1 | >0.9999 |
| EBV-BM+EBV-BR tetramer+ ≥ 0.1 | 0.62 | 0.36 |
| EBV genome copy (log)/10^6^ B cells > 4.5 | 4.5 | 0.043* |

# Fisher’s Exact Test used to calculate relative risk

* indicates this variable increases relative risk of developing severe-AIM diagnosis
